# Association between initial benzodiazepine prescribing patterns and time to benzodiazepine discontinuation: A population-based retrospective cohort study

**DOI:** 10.1371/journal.pmed.1005126

**Published:** 2026-06-18

**Authors:** Nikki Bozinoff, Tanya S. Hauck, Robert A. Kleinman, Matthew E. Sloan, Beth A. Sproule, Simone N. Vigod, Jennifer Wyman, Priscila Pequeno, Tara Gomes

**Affiliations:** 1 Campbell Family Mental Health Research Institute, Centre for Addiction and Mental Health, Toronto, Ontario, Canada; 2 Addictions Division, Centre for Addiction and Mental Health, Toronto, Ontario, Canada; 3 Deparent of Family and Community Medicine, Temerty Faculty of Medicine, University of Toronto, Toronto, Ontario, Canada; 4 Department of Psychiatry, Temerty Faculty of Medicine, University of Toronto, Toronto, Ontario, Canada; 5 Institute of Health Policy, Management and Evaluation, University of Toronto, Toronto, Ontario, Canada; 6 Institute for Mental Health Policy Research, Centre for Addiction and Mental Health, Toronto, Ontario, Canada; 7 Department of Pharmacology & Toxicology, University of Toronto, Toronto, Ontario, Canada; 8 Institute of Medical Science, University of Toronto, Toronto, Ontario, Canada; 9 Department of Psychological Clinical Science, University of Toronto Scarborough, Toronto, Ontario, Canada; 10 Leslie Dan Faculty of Pharmacy, University of Toronto, Toronto, Ontario, Canada; 11 ICES, Toronto, Ontario, Canada; 12 Department of Psychiatry, Women’s College Hospital and Women’s College Hospital Research and Innovation Institute, Toronto, Ontario, Canada; 13 Department of Medicine, Women’s College Hospital, Toronto, Ontario, Canada; 14 Li Ka Shing Knowledge Institute, St. Michael’s Hospital, Toronto, Ontario, Canada; University of New South Wales, AUSTRALIA

## Abstract

**Background:**

Long-term benzodiazepine use has been associated with increased risk of morbidity and mortality. Preventing long-term use through safer prescribing practices has received little attention to date. We sought to better understand associations between initial prescription characteristics and duration of benzodiazepine use.

**Methods and findings:**

This was a retrospective population-based cohort study of 1,820,808 adults in Ontario with incident benzodiazepine prescriptions between January 1, 2013 and December 31, 2020, with follow-up to December 31, 2021. The primary exposure was duration of the index prescription (≤7 days—referent group, 8–14 days, 15–30 days, or >30 days). Secondary exposures were: (a) duration of action of index benzodiazepine(s) prescription (short-acting, long-acting or both); (b) number of benzodiazepine dispensed on index (1 or 2+); and (c) mean daily dose of the index prescription in Diazepam Milligram Equivalents (DMEs). The primary outcome was time to benzodiazepine discontinuation in days. Multivariable models were adjusted for age, sex, anxiety, insomnia, and substance use disorders as well as other important comorbidities and socio-demographic characteristics. The median age at index was 53 years (Interquartile Range (IQR) 38–67), and 62.6% were women. The median time to discontinuation in women was 16 days (IQR: 6–29) while the median time to discontinuation in men was 19 days (IQR: 6–29). Lorazepam was the most commonly prescribed benzodiazepine on index (63.9%), followed by clonazepam (17.3%) and diazepam (5.8%). In multivariable Cox Proportional Hazards Models, longer index prescriptions were associated with a lower likelihood of benzodiazepine discontinuation (adjusted Hazard Ratio (aHR) 0.54 (95% Confidence Interval (CI) [0.54,0.54]) for 8–14 days; aHR 0.26 (95% CI [0.25,0.26] for 15–30 days and aHR 0.14 (95% CI [0.14,0.14]) for >30 days, compared to ≤7 days, respectively). Being prescribed two or more benzodiazepines versus 1 was also associated with a reduced likelihood of discontinuation (aHR 0.59 (95% CI [0.57,0.61])), as was being prescribed long-acting benzodiazepines (aHR 0.80 (95% CI [0.80,0.80])) or a combination of short and long acting benzodiazepine (aHR 0.84 (95% CI [0.80,0.88])) versus short-acting benzodiazepines alone. Mean daily doses of >5 to ≤10 DME and >10 to ≤20 DME were associated with an increased likelihood of discontinuation (aHR 1.03 (95% CI [1.03,1.03]); aHR: 1.03 (95% CI [1.03,1.04])), whereas doses >20 DME were associated with a reduced likelihood of discontinuation (aHR 0.98 (95% CI [0.97,0.98])) compared with ≤5 DME. Findings may be subject to bias from unmeasured confounding.

**Conclusion:**

This large population-based cohort study found that prescribing shorter courses of benzodiazepines, use of a single benzodiazepine, use of a short-acting agent, were associated with reduced likelihood of long-term benzodiazepine use. Findings suggest that simple changes to prescribing practices could reduce prolonged benzodiazepine use and the morbidity and mortality associated with long-term use of these medications.

## Author summary

**Why was this study done?:**

- Benzodiazepines are a class of commonly used anti-anxiety medication that are disproportionately prescribed to women.
- Long-term use of benzodiazepines is generally not recommended because of known harms associated with their use, including addiction, memory problems, increased risk of falls, overdose, and death.
- Previous research suggested that some characteristics of initial prescriptions were associated with prolonged use; however, results were not always consistent, and it was unclear if these factors were the same in men compared with women.
- We therefore sought to better understand the association between initial prescription characteristics and duration of benzodiazepine use, and whether that relationship differed by sex.

**What did the researchers do and find?:**

- This was a retrospective population-based cohort study of 1,820,808 adults in Ontario with new benzodiazepine prescriptions between January 1, 2013 and December 31, 2020 and follow-up to December 31, 2021.
- We examined the association between initial prescription characteristics including duration of the prescription, duration of action of the prescribed benzodiazepine(s) (short-acting, long-acting, or both), number of benzodiazepine dispensed (1 or 2+) and the mean daily dose of the prescription in Diazepam Milligram Equivalents (DMEs), and time to benzodiazepine discontinuation.
- Across multiple analyses, we found that prescribing shorter courses of benzodiazepines, use of a single benzodiazepine and use of a short-acting agent, were associated with reduced likelihood of long-term benzodiazepine use, and these results were consistent across sex.

**What do these findings mean?:**

- Findings suggest that simple changes to prescribing practices including limiting initial prescriptions to <7 days and choosing a single, short-acting agent could reduce prolonged benzodiazepine use and associated morbidity and mortality in both men and women.
- Results may be limited by factors that were not measured in our datasets or considered in our analyses.

## Abstract 3

In this retrospective cohort study, Nikki Bozinoff and colleagues investigate the association between initial prescription characteristics and duration of benzodiazepine use.

## Introduction

Benzodiazepines are commonly prescribed medications—in 2023, over 23 million individuals in the United States reported use of benzodiazepines [1]. Benzodiazepines have a number of therapeutic uses including in the acute management of seizures, alcohol withdrawal and panic, however, chronic use has been implicated in negative health outcomes including increased risk of mortality, falls, cognitive impairment, overdose (particularly when combined with opioids and other sedating agents), motor vehicle collisions, and substance use disorder [2–4]. Women are particularly affected—across multiple countries, women are prescribed benzodiazepines at 1.5–2 times the rate with which they are prescribed to men [5–10]. In this context, efforts to reduce long-term benzodiazepine use have emerged, and include motivational interventions and tapering guidelines/protocols [11–13]; however, in 2018 approximately 50% of individuals prescribed benzodiazepines in the US received them for 2 months or longer [1], and as many as 15% of individuals exposed to benzodiazepines remain on these medications in the long-term [14]. Prevention of long-term use via safer prescribing practices has received relatively little attention to date, and it remains unclear if these safer prescribing factors are the same in men compared with women [14]. Past work has demonstrated an association between ‘heavy’ use during the initiation period (higher frequency, daily use, etc.) and prolonged use [15–17]. A larger number of tablets dispensed initially [18], a longer initial prescription duration in special populations (older adults, individuals with depression) [19,20], and concurrent use of multiple benzodiazepines [21,22] have all been associated with long-term use. Studies examining the association between the type of benzodiazepine prescribed at initiation (short versus long-acting) and long-term use, have had mixed results with some suggesting longer-acting benzodiazepines may be associated with prolonged use [23], others finding short-acting benzodiazepines are associated with prolonged use [17,21], and still others finding no association between benzodiazepine half-life and time to discontinuation [24,25]. In the opioid literature, research investigating initial prescriptions have revealed that longer initial prescriptions, higher initial doses, and use of long-acting versus short-acting formulations are associated with long-term use [26,27], leading to recommendations that clinicians prescribing opioids for acute pain prescribe the lowest effective dose of immediate-release opioids, and in “no greater quantity than needed for the expected duration of pain severe enough to require opioids” [28]. Current benzodiazepine prescribing guidance generally recommends “short-term” prescribing, typically defined as prescriptions lasting less than 4 weeks [29]. However, four weeks remains a relatively long prescription duration, and even shorter durations may be preferable. Further understanding the impact of initial benzodiazepine prescribing factors on sustained benzodiazepine use could help prescribers reduce harm by preventing long-term use and dependence. We therefore set out to understand the association of initial prescription characteristics with time to discontinuation of benzodiazepines in adults 18 years or older in Ontario, Canada, and given the disproportionate impact of benzodiazepine prescribing on women, whether this relationship differed by sex. We hypothesized that longer initial prescription durations would be associated with a reduced likelihood of discontinuation.

## Methods

### Study design and setting

We conducted a retrospective population-based cohort study using linked administrative health data from the province of Ontario, Canada. Ontario is home to over 14 million people who receive publicly-funded healthcare via a single payer, the Ontario government. This study was approved by the Centre for Addiction and Mental Health Research Ethics Board, #048/2022. The dataset creation plan for this study can be viewed in the supplement (S1 Data). The study’s reporting followed the Strengthening the Reporting of Observational Studies in Epidemiology (STROBE) guideline (S1 STROBE Checklist).

### Data sources

Data for this study were held at ICES, an independent, non-profit research institute. We utilized the Narcotic Monitoring System (NMS), a mandatory prescription monitoring system that captures all controlled substances dispensed by a community pharmacy, regardless of payer. This includes all benzodiazepines, opioids, and stimulant prescriptions. Demographic and vital statistics for all Ontarians were captured via the Registered Persons Database (RPDB), physician services via the Ontario Health Insurance Plan (OHIP) and physician characteristics via the Corporate Provider Database and the ICES Physician Database. We characterized inpatient hospitalizations via the Discharge Abstract Database (DAD) [30], mental health hospitalizations using the Ontario Mental Health Reporting System, and emergency department visits via the National Ambulatory Care Reporting System (NACRS). These databases were linked using unique encoded identifiers and analyzed at ICES. More information about the included databases are available in S1 Appendix, Table A.

### Cohort creation

We included adults aged 18 years or older with a new prescription for oral benzodiazepines (excluding z-drugs which are not uniformly available in the NMS database) between January 1, 2013 and December 31, 2020, defined as no benzodiazepine prescription in the 182 days prior to cohort entry. Episodes where midazolam or clobazam were prescribed were excluded as these benzodiazepines are typically used for specific indications related to palliative care or seizure disorders. We also excluded individuals who had received palliative care services (including hospice services) in the 6 months prior to cohort entry as this population may have unique considerations with respect to medication continuation at end-of-life. We excluded individuals with missing age or sex, age <18 or >105 years, those who were not Ontario residents, and episodes with death date prior to index date. We then constructed episodes of continuous benzodiazepine use on the basis of receiving a refill within 1.5 times the days supply of the prior benzodiazepine prescription, with a minimum look-forward window of 30 days. If two benzodiazepine prescriptions were overlapping, we assumed they were taken concurrently rather than sequentially. The prescription end date was the date on which the last prescription was dispensed plus the days supplied, subtracting 1 (to account for us of medication on day of dispense). As more than one episode per individual could meet inclusion criteria, and we randomly included one episode per individual for the primary analysis. In a first-episode sensitivity analysis, we included only the first episode in the accrual period. In recurrent event sensitivity analyses, we utilized a 2-year look back for no benzodiazepine use, and accrued episodes from July 1, 2014 to December 31, 2020.

### Exposures

The primary exposure was the duration of benzodiazepine use in days categorized as ≤7 days (referent), 8–14 days, 15–30 days, and >30 days. If more than one prescription was dispensed on index, we categorized the prescription with the longest duration. Secondary exposures were duration of action of benzodiazepine(s) dispensed on index defined as short-acting, long-acting or both short- and long-acting (see Table B in S1 Appendix for classification used); whether one or more than one benzodiazepine was dispensed on index; and the mean daily dose of the index prescription, in Diazepam Milligram Equivalents (DMEs) [31]. To calculate the mean daily dose of the index prescription in DMEs, we first calculated the total DME based on drug name and strength of benzodiazepine (benzodiazepine strength × Conversion Factor = DMEs). We then calculated the mean daily dose as strength in DME * quantity dispensed/days supplied. If more than one prescription was dispensed at index, their mean daily DMEs were summed (See Table C in S1 Appendix for conversion factors used). In all cases, categories were defined to align with standard clinical practice as it relates to benzodiazepine prescribing.

### Outcome

The outcome of interest was time to benzodiazepine discontinuation as defined above (no new benzodiazepine dispense within 1.5× the days supplied of the last prescription, or a minimum of 30 days). We censored on the first of death, 3 years from cohort entry, or maximum follow up (December 31, 2021).

### Covariates

Covariates were measured at baseline and were selected ‘a priori’ based on clinical expertise and their known or hypothesized relationship with the exposure and outcome. We included demographic variables (age, sex, rurality of residence, and neighborhood income quintile), concurrent mental health and substance use disorders (alcohol use disorder [32], harmful sedative-hypnotic use or dependence, receipt of opioid agonist therapy (OAT) in the last 6 months, as well as psychotic disorders [33], anxiety and mood disorders [33] and insomnia) [34] and medical comorbidity by using the Johns Hopkins Adjusted Clinical Groups (ACG) System Version 10 [35]. Patients were assigned a weighted score based on presence or absence of 32 ACG System Aggregated Diagnosis Groups (ADGs) characterizing medical conditions based on their use of inpatient and outpatient healthcare services in the preceding 2 years. ADGs were summed and grouped into quintiles [35]. Finally, we also included the medical discipline of the prescriber of the index prescription (see Table D in S1 Appendix for full variable definitions).

### Analysis

Baseline characteristics (see Table E in S1 Appendix for full variable definitions) were stratified by sex and compared using standardized differences. A standardized difference >0.10 was considered meaningful [36]. Kaplan–Meier curves were used to present crude survival probabilities for the primary outcome, and the Log-Rank test was used to test for differences in survival functions between strata. We used multivariable Cox proportional hazards regression modeling to characterize the association between the exposure variables and our primary outcome, while adjusting for the covariates described above. We tested the proportional hazards assumption via visual inspection of log-negative-log survival curves (Figs A–D in S1 Appendix). In a secondary analysis, we stratified models by sex. Two pre-specified sensitivity analyses were conducted in order to assess the robustness of our findings to methodologic assumptions made in the primary analysis related to the definition of continuous use episodes and the calculation of DMEs. In the first sensitivity analysis, we relaxed the definition of discontinuation by looking forward 2 times the days supplied, with a minimum look forward of 60 days for the next benzodiazepine prescription. In a second sensitivity analysis, we used an alternative published conversion factor to calculate DMEs [37] (Table C in S1 Appendix). Notable differences in the conversion factors for DME calculations included differences in the equivalence of lorazepam (1 mg lorazepam = 10 mg diazepam in the main analysis versus 2 mg lorazepam = 10 mg in the sensitivity analysis), alprazolam (0.5 mg alprazolam = 10 mg diazepam in the main analysis versus 1 mg alprazolam = 10 mg diazepam in the sensitivity analysis) and clonazepam (0.5 mg clonazepam = 10 mg diazepam in the main analysis versus 1 mg clonazepam = 10 mg diazepam in the sensitivity analysis). In order to ensure there were not systematic changes in prescribing practices over the study period that would impact study results, we also undertook a post-hoc sensitivity analysis stratifying the results by time period (2013–2016 versus 2017–2020) and a sensitivity analysis including only first episodes in the accrual period. Finally, in order to understand the conditional, event-ordered-average effect across recurrent episodes, and whether prescribing factors associated with prolonged benzodiazepine use changed with recurrent prescription episodes, we conducted post-hoc recurrent event analyses using the Prentice–Williams–Peterson Total Time model overall, and stratified by episode number. This model estimates the exposures’ effects of the hazard of a recurrent event, conditional on prior event history [38]. The analyses were completed using SAS version 9.3, and a 2-tailed *p* value of <0.05 was deemed significant. There was missingness in only one variables (specialty of physician prescriber), and this was grouped separately.

## Results

**Fig 1:**
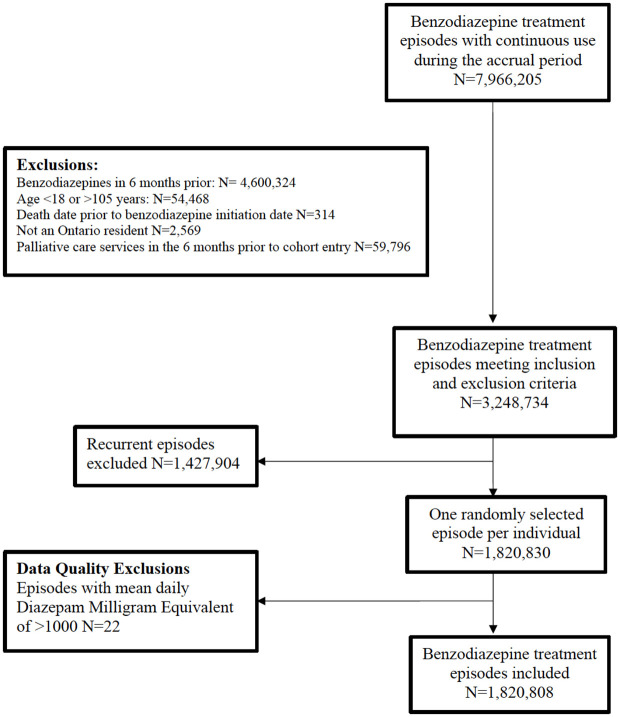
Cohort assembly flowchart.

Overall, 3,248,734 benzodiazepine index prescriptions met inclusion criteria. Following the random inclusion of one index prescription per individual, 1,820,808 episodes were included in the analysis (Fig 1).

Baseline characteristics overall and stratified by sex are presented in Table 1. Overall, the median age at index was 53 years (Interquartile Range (IQR) 38–67 years), and 62.6% of included episodes occurred in females. Males were more likely to have a diagnosis of alcohol use disorder (6.7% versus 2.3%) and any substance use disorder (5.4% versus 2.0%) while females were more likely to have a mood or anxiety disorder diagnosis (46.5% versus 41.0%). Lorazepam was the most commonly prescribed benzodiazepine on index overall (63.9%), followed by clonazepam (17.3%) and diazepam (5.8%) (Table 1). Males were more likely to be prescribed diazepam (9.2%) while females were more likely to be prescribed lorazepam (66.9%). Median time to discontinuation overall was 19 days (IQR: 6–29). In women median time to discontinuation was 16 days (IQR: 6–29) while the median time to discontinuation in males was 19 days (IQR: 6–29).

**Table 1:** Baseline characteristics of individuals prescribed benzodiazepines at index, stratified by sex.

Variable	Total *N* = 1,820,808 (%)	Male *N* (%) *n* = 680,403 (37.4%)	Female *N* (%) *n* = 1,140,405 (62.6%)	Standard difference
**Age in years (median, IQR)**	53 (38–67)	53 (38–67)	53 (38–67)	0.023
**Location of Residence**				
Urban	1,626,265 (89.3%)	606,447 (89.1%)	1,019,818 (89.4%)	0.01
Rural	194,543 (10.7%)	73,956 (10.9%)	120,587 (10.6%)	0.01
**Income quintile**				
1st (lowest)	379,136 (20.8%)	140,872 (20.7%)	238,264 (20.9%)	0.005
2nd	368,753 (20.3%)	136,688 (20.1%)	232,065 (20.3%)	0.006
3rd	363,401 (20.0%)	135,821 (20.0%)	227,580 (20.0%)	0
4th	352,694 (19.4%)	132,050 (19.4%)	220,644 (19.3%)	0.002
5th	356,824 (19.6%)	134,972 (19.8%)	221,852 (19.5%)	0.01
**Residing in long-term care**	56,562 (3.1%)	19,037 (2.8%)	37,525 (3.3%)	0.029
**Concurrent opioid prescription**	223,487 (12.3%)	92,855 (13.6%)	130,632 (11.5%)	0.066
**Alcohol use disorder**	72,159 (4.0%)	45,651 (6.7%)	26,508 (2.3%)	0.212^*^
**Harmful sedative-hypnotic use or dependence**	4,700 (0.3%)	1,834 (0.3%)	2,866 (0.3%)	0.004
**Harmful stimulant use or dependence**	6,308 (0.3%)	3,936 (0.6%)	2,372 (0.2%)	0.059
**Other medications prescribed in the last 6 months**				
Stimulants	28,596 (1.6%)	12,903 (1.9%)	15,693 (1.4%)	0.041
Non-opioid agonist therapy opioid	385,328 (21.2%)	147,106 (21.6%)	238,222 (20.9%)	0.018
Opioid agonist therapy	19,236 (1.1%)	11,319 (1.7%)	7,917 (0.7%)	0.09
Barbiturates	1,473 (0.1%)	382 (0.1%)	1,091 (0.1%)	0.014
Tetrahydrocannabinol products	8,926 (0.5%)	3,483 (0.5%)	5,443 (0.5%)	0.005
**Outpatient mental health visits**				
Psychotic disorders	30,983 (1.7%)	17,081 (2.5%)	13,902 (1.2%)	0.096
Anxiety and mood disorders	809,520 (44.5%)	278,933 (41.0%)	530,587 (46.5%)	0.112^*^
Substance use	60,058 (3.3%)	37,032 (5.4%)	23,026 (2.0%)	0.181^*^
Behavioral and neurodevelopmental disorders	16,091 (0.9%)	8,653 (1.3%)	7,438 (0.7%)	0.064
**ED visit or hospitalization for any mental health or addictions**	114,440 (6.3%)	47,839 (7.0%)	66,601 (5.8%)	0.049
**Insomnia**	191,329 (10.5%)	77,978 (11.5%)	113,351 (9.9%)	0.049
**COPD**	248,174 (13.6%)	101,387 (14.9%)	146,787 (12.9%)	0.059
**Diabetes**	285,595 (15.7%)	123,464 (18.1%)	162,131 (14.2%)	0.107^*^
**HIV**	4,758 (0.3%)	3,839 (0.6%)	919 (0.1%)	0.085
**Specialty of physician prescriber**				
Family Medicine	1,387,951 (76.2%)	505,239 (74.3%)	882,712 (77.4%)	0.074
Psychiatry	126,326 (6.9%)	52,400 (7.7%)	73,926 (6.5%)	0.047
Internal Medicine	25,218 (1.4%)	11,147 (1.6%)	14,071 (1.2%)	0.034
Neurology	15,470 (0.8%)	6,276 (0.9%)	9,194 (0.8%)	0.013
Obstetrics and Gynecology	11,956 (0.7%)	344 (0.1%)	11,612 (1.0%)	0.133^*^
Emergency Medicine	12,691 (0.7%)	6,456 (0.9%)	6,235 (0.5%)	0.047
Other	134,354 (7.4%)	61,076 (9.0%)	73,278 (6.4%)	0.096
Missing	106,842 (5.9%)	37,465 (5.5%)	69,377 (6.1%)	0.025
**Health system utilization in 1 year prior to index date (mean, SD)**				
Number of physician visits	9.64 (9.41)	9.27 (9.72)	9.86 (9.21)	0.063
Number of ED visits	0.95 (2.15)	1.04 (2.33)	0.90 (2.03)	0.062
Number of hospitalizations	0.18 (0.59)	0.21 (0.66)	0.16 (0.55)	0.081
**ADG quintiles**				
0	297,490 (16.3%)	145,093 (21.3%)	152,397 (13.4%)	0.211^*^
1	517,045 (28.4%)	199,973 (29.4%)	317,072 (27.8%)	0.035
2	351,858 (19.3%)	118,663 (17.4%)	233,195 (20.4%)	0.077
3	281,225 (15.4%)	90,654 (13.3%)	190,571 (16.7%)	0.095
4	373,190 (20.5%)	126,020 (18.5%)	247,170 (21.7%)	0.079
**Benzodiazepine prescribed on index**				
ALPRAZOLAM	69,258 (3.8%)	25,184 (3.7%)	44,074 (3.9%)	0.009
BROMAZEPAM	11,750 (0.6%)	4,336 (0.6%)	7,414 (0.7%)	0.002
CHLORDIAZEPOXIDE HCL	5,130 (0.3%)	2,627 (0.4%)	2,503 (0.2%)	0.03
CHLORDIAZEPOXIDE HCL & CLIDINIUM BROMIDE	9,055 (0.5%)	3,648 (0.5%)	5,407 (0.5%)	0.009
CLONAZEPAM	315,064 (17.3%)	122,164 (18.0%)	192,900 (16.9%)	0.027
CLORAZEPATE DIPOTASSIUM	913 (0.1%)	335 (0.0%)	578 (0.1%)	0.001
DIAZEPAM	104,876 (5.8%)	62,261 (9.2%)	42,615 (3.7%)	0.222^*^
FLURAZEPAM HYDROCHLORIDE	3,025 (0.2%)	1,418 (0.2%)	1,607 (0.1%)	0.016
LORAZEPAM	1,162,662 (63.9%)	399,707 (58.7%)	762,955 (66.9%)	0.169^*^
NITRAZEPAM	8,017 (0.4%)	3,626 (0.5%)	4,391 (0.4%)	0.022
OXAZEPAM	49,954 (2.7%)	20,348 (3.0%)	29,606 (2.6%)	0.024
TEMAZEPAM	60,068 (3.3%)	27,514 (4.0%)	32,554 (2.9%)	0.065
TRIAZOLAM	21,036 (1.2%)	7,235 (1.1%)	13,801 (1.2%)	0.014

* Standard difference >0.1.

ED, Emergency Department; SD, Standard Deviation; COPD, Chronic Obstructive Pulmonary Disease; HIV, Human Immunodeficiency Virus; ADG, Aggregated Diagnosis Groups.

**Fig 2:**
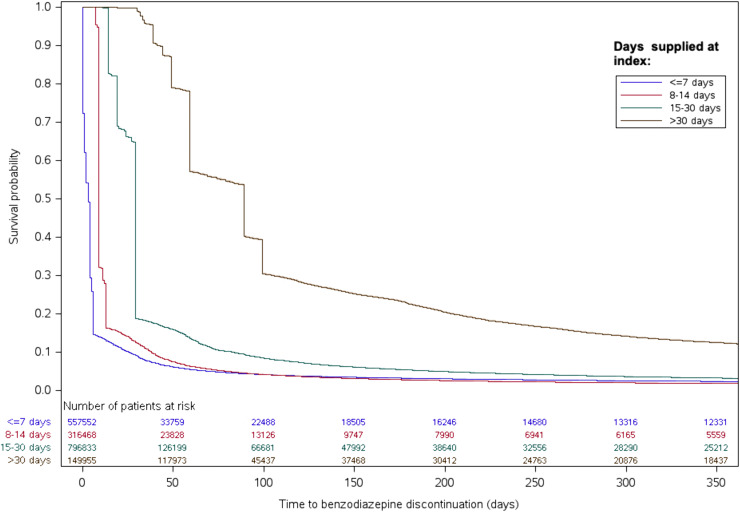
Time to benzodiazepine discontinuation by initial prescription duration (log-rank test, *p* < 0.0001).

Among the 1,820,808 included episodes in the primary analysis, 17,973 (0.99%) were censored due to death, 14,488 (0.80%) were censored at end of follow-up, and 1,788,347 (98.22%) experienced the outcome of interest (discontinuation). In crude models, median time to discontinuation differed by duration of index prescription (median 3 days for initial days supply ≤7 versus 88 days for initial days supply >30). Kaplan-Meier curves are presented in Fig 2 (log-rank test, *p* < 0.0001).

After multivariable adjustment, longer index prescriptions were associated with a reduced hazard of benzodiazepine discontinuation (8–14 days versus ≤7 days adjusted Hazard Ratio [aHR] 0.54 (95% Confidence Interval [CI] [0.54,0.40]; 15–30 days versus ≤7 days aHR 0.26 (95% CI [0.26,0.58]); > 30 days versus ≤7 days, aHR 0.14 (95% CI [0.14,0.14])) (Table 2). Being dispensed both a short-acting and long-acting benzodiazepine together (aHR 0.84 (95% CI [0.80,0.88])) and being prescribed long-acting benzodiazepines alone (aHR 0.80 (95% CI [0.80,0.80])) were associated with a reduced hazard of discontinuation compared to being dispensed short-acting benzodiazepines alone. Being initially prescribed two or more benzodiazepines compared with one was also associated with a reduced hazard of discontinuation (aHR 0.59 (95% CI [0.57,0.61])). Finally, mean daily doses between 5 and ≤10 DME (aHR 1.03 (95% CI [1.03,1.03])) and between 10 and ≤20 DME (aHR 1.03 (95% CI [1.03,1.04])) were associated with an increased hazard of discontinuation compared to doses ≤5 DME, while doses >20 were associated with a reduced hazard of discontinuation (aHR 0.98 (95% CI [0.97,0.98])). In the analysis stratified by sex, results were consistent with the primary analysis with respect to the impact of days supplied at index, the type of benzodiazepine dispensed at index, and the number of benzodiazepines dispensed at index although there were some differences in the direction of the association with the mean daily dose (Table 2). Among females, receipt of all dose categories had an increased hazard of discontinuation compared with ≤5 DME (aHR 1.03 (95% CI [1.03,1.04]), aHR 1.07 (95% CI [1.06, 1.07]), and aHR 1.01 (95% CI [1.00, 1.01]) for >5 - ≤10 DME, > 10-≤20 DME, and >20 DME, respectively, compared with ≤5 DME). Among males, receipt of >5 to ≤10 compared with ≤5 DMEs was associated with an increased hazard of discontinuation (aHR 1.03 (95% CI [1.02, 1.03])), while receipt of doses >10 to ≤20 and >20 DME were associated with a reduced hazard of discontinuation compared to receipt of <5 DMEs (aHR 0.98 (95% CI [0.92, 0.98]), aHR 0.94 (95% CI [0.93, 0.95]), respectively) (Table 2).

**Table 2:** Multivariable Cox proportional hazards model of prescription factors associated with time to benzodiazepine discontinuation overall and stratified by sex.

	Overall					Females	Males
	*N* individuals	*N* outcomes	Outcome per 100 person-years	Unadjusted HR (95% CI)	aHR (95% CI)	aHR (95% CI)	aHR (95% CI)
**Days supplied at index**							
≤7 days	557,552	547,947	1346.62	1.00	1.00	1.00	1.00
8–14 days	316,468	312,399	1163.42	0.57 (0.57, 0.58)	0.54 (0.54, 0.54)	0.54 (0.54, 0.54)	0.54 (0.53, 0.54)
15–30 days	796,833	783,483	611.75	0.27 (0.27, 0.27)	0.26 (0.25, 0.26)	0.25 (0.25, 0.25)	0.27 (0.26, 0.27)
>30 days	149,955	144,518	200.06	0.15 (0.15, 0.15)	0.14 (0.14, 0.14)	0.14 (0.14, 0.14)	0.15 (0.15, 0.15)
**Type of benzodiazepine dispensed at index**							
Short-acting	1,374,245	1,351,629	783.52	1.00	1.00	1.00	1.00
Short- and long-acting	5,249	4,858	190.99	0.49 (0.48, 0.51)	0.84 (0.80-0.88)	0.92 (0.86, 0.98)	0.75 (0.70, 0.81)
Long-acting	441,314	431,860	465.36	0.77 (0.77, 0.77)	0.80 (0.80-0.80)	0.76 (0.75, 0.76)	0.86 (0.85, 0.86)
**Number of benzodiazepines dispensed at index**							
1	1,812,898	1,780,999	674.47	1.00	1.00	1.00	1.00
≥2	7,910	7,348	193.80	0.52 (0.51, 0.54)	0.59 (0.57, 0.61)	0.55 (0.52, 0.58)	0.65 (0.61, 0.69)
**Mean daily dose of index prescription in Diazepam Milligram Equivalents**							
≤5	352,254	347,293	711.17	1.00	1.00	1.00	1.00
>5 to ≤10	790,305	778,587	663.46	1.03 (1.02, 1.03)	1.03 (1.03, 1.03)	1.03 (1.03, 1.04)	1.03 (1.02, 1.03)
>10 to ≤20	440,027	431,908	632.47	1.14 (1.14, 1.15)	1.03 (1.03, 1.04)	1.07 (1.06, 1.07)	0.98 (0.98, 0.99)
>20	238,222	230,559	690.82	1.43 (1.42, 1.43)	0.98 (0.97, 0.98)	1.01 (1.00, 1.01)	0.94 (0.93, 0.95)

HR, Hazard Ratio; 95% CI, 95% Confidence Interval; aHR, adjusted Hazard Ratio.

Adjusted models were adjusted for age, rurality of residence, income quintile, alcohol use disorder, harmful sedative-hypnotic use or dependence, receipt of opioid agonist therapy in the last 6 months, psychotic disorders, anxiety and mood disorders, insomnia, discipline of prescriber, and Aggregated Diagnosis Groups. Overall model was also adjusted for sex.

In the sensitivity analysis loosening the definition of discontinuation to allow a longer look forward, results were consistent (Table 3) with the exception that doses between 10 and ≤20 DME were associated with reduced hazard of discontinuation (aHR 0.96 (95% CI [0.96,0.97])) compared with ≤5 DME.

**Table 3:** Sensitivity analysis with 2x days supply look forward and minimum look-forward of 60 days. Multivariable Cox proportional hazards model of prescription factors associated with time to benzodiazepine discontinuation.

	*N* individuals	*N* outcomes	Outcome per 100 person-years	Unadjusted Hazard Ratio (95% CI)	Adjusted Hazard Ratio (95% CI)
**Days supplied at index**					
≤7 days	557,777	547,947	984.44	1.00	1.00
8–14 days	316,369	310,274	744.40	0.58 (0.58, 0.58)	0.55 (0.54, 0.55)
15–30 days	796,927	774,782	421.02	0.32 (0.32, 0.32)	0.30 (0.30, 0.31)
>30 days	147,449	137,533	136.76	0.18 (0.18, 0.18)	0.17 (0.17, 0.17)
**Type of benzodiazepine dispensed at index**					
Short-acting	1,372,823	1,339,650	544.94	1.00	1.00
Short- and long-acting	5,176	4,639	143.27	0.49 (0.48, 0.51)	0.88 (0.84, 0.92)
Long-acting	440,523	424,078	319.71	0.76 (0.76, 0.77)	0.82 (0.82, 0.82)
**Number of benzodiazepines dispensed at index**					
1	1,810,666	1,761,312	467.40	1.00	1.00
≥2	7,856	7,055	144.39	0.53 (0.51, 0.54)	0.65 (0.62, 0.67)
**Mean daily dose of index prescription in Diazepam Milligram Equivalents**					
≤5	351,872	344,549	529.82	1.00	1.00
>5 to ≤10	788,631	769,918	469.40	1.01 (1.00, 1.01)	1.00 (1.00, 1.01)
>10 to ≤20	439,723	426,289	418.63	1.06 (1.06, 1.07)	0.96 (0.96, 0.97)
>20	238,296	227,611	447.77	1.26 (1.25, 1.27)	0.87 (0.87, 0.88)

95% CI, 95% Confidence Interval.

Adjusted models were adjusted for age, rurality of residence, sex, income quintile, alcohol use disorder, harmful sedative-hypnotic use or dependence, receipt of opioid agonist therapy in the last 6 months, psychotic disorders, anxiety and mood disorders, insomnia, discipline of prescriber, and Aggregated Diagnosis Groups.

In the sensitivity analysis using a different DME conversion factor, the direction of the effects were similar except for daily doses between 10 and ≤20 DME were associated with a reduced hazard of discontinuation (aHR 0.95 (95% CI [0.95,0.96])), while daily doses between 5 and ≤10 and >20 DME were associated with higher hazard of discontinuation (aHR 1.06 (95% CI [1.05–1.06]); aHR 1.02 (95% CI [1.01,1.03]), respectively; Table 4).

**Table 4:** Sensitivity analysis of multivariable Cox proportional hazards model of prescription factors associated with time to benzodiazepine discontinuation using alternate diazepam milligram equivalent conversion.

	*N* individuals	*N* outcomes	Outcome per 100 person-years	Unadjusted Hazard Ratio (95% CI)	Adjusted Hazard Ratio (95% CI)
**Days supplied at index**					
≤7 days	557,552	547,947	1346.62	1.00	1.00
8–14 days	316,468	312,399	1163.42	0.57 (0.57, 0.58)	0.54 (0.54, 0.54)
15–30 days	796,833	783,483	611.75	0.27 (0.27, 0.27)	0.26 (0.26, 0.26)
>30 days	149,955	144,518	200.06	0.15 (0.15, 0.15)	0.14 (0.14, 0.14)
**Type of benzodiazepine dispensed at index**					
Short-acting	1,374,245	1,351,629	783.52	1.00	1.00
Short- and long-acting	5,249	4,858	190.99	0.49 (0.48, 0.51)	0.83 (0.79, 0.88)
Long-acting	441,314	431,860	465.36	0.77 (0.77, 0.77)	0.79 (0.79, 0.80)
**Number of benzodiazepines dispensed at index**					
1	1,812,898	1,780,999	674.47	1.00	1.00
≥2	7,910	7,348	193.80	0.52 (0.51, 0.54)	0.58 (0.56, 0.61)
**Mean daily dose of index prescription in Diazepam Milligram Equivalents**					
≤5	1,045,609	1,030,615	685.06	1.00	1.00
>5 to ≤10	483,192	474,658	658.93	1.15 (1.14, 1.15)	1.06 (1.05, 1.06)
>10 to ≤20	217,594	211,951	603.30	1.21 (1.20, 1.21)	0.95 (0.95, 0.96)
>20	74,413	71,123	694.43	1.60 (1.59, 1.62)	1.02 (1.01, 1.03)

Adjusted models were adjusted for age, sex, rurality of residence, income quintile, alcohol use disorder, harmful sedative-hypnotic use or dependence, receipt of opioid agonist therapy in the last 6 months, psychotic disorders, anxiety and mood disorders, insomnia, discipline of prescriber, and Aggregated Diagnosis Groups.

Similarly, in both post-hoc sensitivity analyses stratifying the results by time period (2013–2016 versus 2017–2020) (Table F in S1 Appendix) and the sensitivity analysis including the first episode of benzodiazepines per individual during the accrual period (Table G in S1 Appendix), results were consistent with the primary analysis with respect to the impact of days supplied at index, type of benzodiazepine at index, and the number of benzodiazepines dispensed at index, although there were some differences in the direction of the association with the mean daily dose.

Finally, in the overall recurrent event analysis which included multiple episodes per individual (Table 5, *N* = 2,380,122 episodes), results were consistent with the primary model, except that compared with mean daily doses ≤5 DME, all higher dose categories were associated with a reduced likelihood of discontinuation (aHR 0.99 (95% CI [0.98,0.99]), aHR 0.98 (95% CI [0.98, 0.99]), aHR 0.98 (95% CI [0.97,0.99]), for doses >5 to ≤10 DME, >10 to ≤20 DME, and >20 DME, respectively). In the recurrent event model stratified by episode number (Table 6), the effect of the exposure variables were of the same direction and similar magnitude of effect across episode number except for mean daily dose, where the protective effect of lower mean daily doses was consistent in earlier episodes, but not for an individuals’ 4th or greater episode.

**Table 5:** Recurrent event sensitivity analysis using PWP-TT model (*N* = 2,380,122 episodes).

	Unadjusted Hazard Ratio (95% CI)	Adjusted Hazard Ratio (95% CI)
**Days supplied at index**		
≤7 days	1.00	1.00
8–14 days	0.81 (0.80, 0.82)	0.73 (0.72, 0.74)
15–30 days	0.40 (0.39, 0.40)	0.36 (0.35, 0.36)
>30 days	0.16 (0.16, 0.16)	0.14 (0.14, 0.14)
**Type of benzodiazepine dispensed at index**		
Short-acting	1.00	1.00
Short- and long-acting	0.46 (0.44, 0.48)	0.78 (0.72, 0.84)
Long-acting	0.76 (0,76, 0.77)	0.80 (0.79, 0.80)
**Number of benzodiazepines dispensed at index**		
1	1.00	1.00
≥2	0.51 (0.50, 0.53)	0.62 (0.59, 0.66)
**Mean daily dose of index prescription in Diazepam Milligram Equivalents**		
≤5	1.00	1.00
>5 to ≤10	0.99 (0.98, 0.99)	0.99 (0.98, 0.99)
>10 to ≤20	1.06 (1.06, 1.07)	0.98 (0.98, 0.99)
>20	1.30 (1.29, 1.32)	0.98 (0.97, 0.99)

PWP-TT, Prentice–Williams–Peterson Total Time model; 95% CI, 95% Confidence Interval.

Adjusted models were adjusted for age, sex, rurality of residence, income quintile, alcohol use disorder, harmful sedative-hypnotic use or dependence, receipt of opioid agonist therapy in the last 6 months, psychotic disorders, anxiety and mood disorders, insomnia, discipline of prescriber, and Aggregated Diagnosis Groups.

**Table 6:** Recurrent event sensitivity analysis using PWP-TT model, stratified by episode number.

	Unadjusted Hazard Ratio (95% Confidence Interval)				Adjusted Hazard Ratio (95% Confidence Interval)			
	Episode 1 *N* = 1,385,160	Episode 2 *N* = 526,961	Episode 3 *N* = 248,836	Episode ≥4 *N* = 219,165	Episode 1 *N* = 1,385,160	Episode 2 *N* = 526,961	Episode 3 *N* = 248,836	Episode ≥4 *N* = 219,165
**Days supplied at index**								
≤7 days	1.00	1.00	1.00	1.00	1.00	1.00	1.00	1.00
8–14 days	0.70 (0.70, 0.71)	1.05 (1.02, 1.09)	1.09 (1.03, 1.15)	1.05 (0.98, 1.12)	0.65 (0.65, 0.66)	0.89 (0.86, 0.92)	0.90 (0.86, 0.95)	0.92 (0.86, 0.99)
15–30 days	0.32 (0.32, 0.32)	0.57 (0.56, 0.58)	0.58 (0.55, 0.61)	0.50 (0.47, 0.53)	0.30 (0.30, 0.30)	0.47 (0.46, 0.48)	0.47 (0.45, 0.49)	0.44 (0.42, 0.47)
>30 days	0.17 (0.17, 0.17)	0.19 (0.18, 0.19)	0.18 (0.17, 0.19)	0.16 (0.15, 0.16)	0.16 (0.16, 0.16)	0.15 (0.14, 0.15)	0.14 (0.13, 0.15)	0.14 (0.13, 0.15)
**Type of benzodiazepine dispensed at index**								
Short-acting	1.00	1.00	1.00	1.00	1.00	1.00	1.00	1.00
Short- and long-acting	0.51 (0.49, 0.52)	0.35 (0.32, 0.38)	0.44 (0.39, 0.50)	0.59 (0.50, 0.69)	0.81 (0.75, 0.86)	0.75 (0.62, 0.89)	0.74 (0.57, 0.95)	0.83 (0.63, 1.08)
Long-acting	0.79 (0.78, 0.79)	0.70 (0.69, 0.71)	0.75 (0.73, 0.76)	0.80 (0.78, 0.81)	0.81 (0.81,0.81)	0.75 (0.74, 0.76)	0.81 (0.79, 0.82)	0.85 (0.84, 0.87)
**Number of benzodiazepines dispensed at index**								
1	1.00	1.00	1.00	1.00	1.00	1.00	1.00	1.00
≥2	0.54 (0.53, 0.56)	0.41 (0.39, 0.44)	0.53 (0.48, 0.58)	0.63 (0.56, 0.71)	0.65 (0.61, 0.68)	0.54 (0.47, 0.63)	0.69 (0.56, 0.85)	0.67 (0.56, 0.81)
**Mean daily dose of index prescription in Diazepam Milligram Equivalents**								
≤5	1.00	1.00	1.00	1.00	1.00	1.00	1.00	1.00
>5 to ≤10	0.97 (0.97, 0.98)	0.97 (0.95, 0.98)	1.01 (0.99, 1.03)	1.10 (1.08, 1.12)	0.99 (0.98,0.99)	0.97 (0.96,0.99)	0.98 (0.96,1.00)	1.01 (0.99,1.02)
>10 to ≤20	1.06 (1.05, 1.06)	0.99 (0.97, 1.00)	1.08 (1.05, 1.11)	1.26 (1.22, 1.29)	0.99 (0.98,0.99)	0.94 (0.92,0.96)	0.97 (0.94,1.00)	1.05 (1.02,1.08)
>20	1.34 (1.33, 1.35)	1.12 (1.09, 1.15)	1.27 (1.22, 1.31)	1.61 (1.54, 1.68)	0.98 (0.97,0.99)	0.93 (0.91,0.96)	0.97 (0.93,1.01)	1.09 (1.04,1.15)

PWP TT model, Prentice–Williams–Peterson Total Time model.

Adjusted models were adjusted for age, sex, rurality of residence, income quintile, alcohol use disorder, harmful sedative-hypnotic use or dependence, receipt of opioid agonist therapy in the last 6 months, psychotic disorders, anxiety and mood disorders, insomnia, discipline of prescriber, and Aggregated Diagnosis Groups.

## Discussion

In this analysis of nearly 2 million benzodiazepine treatment episodes, after adjusting for potential confounding variables, several initial benzodiazepine prescribing patterns were associated with time to benzodiazepine discontinuation. Specifically, longer initial index prescriptions were strongly associated with a reduced likelihood of benzodiazepine discontinuation, a finding that was replicated in sex stratified analyses, and in all sensitivity analyses. Furthermore, receipt of long-acting benzodiazepines at initiation, multiple benzodiazepine types initiated, two or more benzodiazepines initiated, were consistently associated with prolonged benzodiazepine use across the analyses. Across the various models, the aHR of mean daily dose was close to 1, suggesting mean daily dose is a less important initial prescribing factor. Effect sizes were largest for longer initial prescriptions, and 2 or more benzodiazepines (compared with 1) initiated suggesting these may be clinically more important factors associated with long-term use.

Our findings replicate what has been found in the opioid literature [26], which has led to recommendations of shorter initial prescription durations for managing acute pain following surgery or ED visits. Existing epidemiologic studies in specific populations (older adults [19], individuals with depression and concurrent anti-depressant use [20]) have also found an association between initial benzodiazepine prescription duration and long-term use. Current benzodiazepine prescribing guidance generally recommends “short-term” prescribing, however, this is typically defined as prescriptions lasting less than 4 weeks [29]. Our findings suggest that the risk of sustained benzodiazepine use is increased with prescriptions lasting greater than 7 days, which suggests that this recommendation is too liberal.

Unsurprisingly, provision of 2 or more benzodiazepines at time of initiation was associated with a reduced hazard of discontinuation, a finding that was also consistent across males and females and robust to changes in definition of discontinuation. Similar findings were observed in a population-based observational cohort of individuals prescribed benzodiazepines in Taiwan which found that being prescribed 2 or ≥3 benzodiazepines increased the odds of long-term use [21]. Similarly, prescription of a short-acting plus a long-acting agent compared with short-acting alone was associated with a reduced likelihood of discontinuation. Given that there are few clinical scenarios where simultaneous prescription of more than one benzodiazepine is necessary or indicated, prescription of more than one agent should be avoided.

As in the opioid literature, we also found that the use of long-acting agents at initiation was associated with a reduced likelihood of discontinuation compared with short-acting only [26]. Some observational studies of people who use benzodiazepines have concurred that longer-acting benzodiazepines may be associated with prolonged use [23], although elsewhere, short-acting agents have been associated with prolonged use [17,21], or no association has been found between benzodiazepine half-life and prolonged use [24,25]. Tolerance may be achieved more quickly with long-acting formulations leading to physiologic dependence and therefore continued use. High-potency and rapid-onset benzodiazepines, can enhance reinforcing subjective effects and have been associated with dose escalation and long-term use [14,16,18]. Further research to disentangle the effect of index prescription potency, time to onset, and half-life would be an important future direction. Overall, our findings suggest that the use of a single, short-acting agent may reduce the risk of prolonged benzodiazepine use.

In our primary analysis, among males, and in a sensitivity analysis with a longer look forward, we found that those dispensed index prescriptions with daily doses >20 DME compared with ≤5 DME, were more likely to remain on benzodiazepines, although this was not consistent across the other sensitivity analyses. Interestingly, in the recurrent event analysis stratified by episode number, doses ≤5 DME were protective against prolonged use, but this effect disappeared by episode ≥4, suggesting that lower doses may be more important for an individual’s earlier exposures to benzodiazepines. It should be noted however, that all estimates were close to 1.0, limiting clinical significance of these findings, and suggesting that future work should continue to explore the relationship between dose, indication for benzodiazepine use, sex and duration of use. It may be helpful for prescribers to familiarize themselves with DMEs in the same way that morphine equivalents have been widely utilized clinically in opioid prescribing and deprescribing, and they should be aware that higher doses, particularly during an individual’s first benzodiazepine exposures, may be associated with prolonged use.

Our study also adds to the literature, by demonstrating that several prescribing factors (longer days supplied, receipt of two or more benzodiazepines at index, receipt of long-acting or both short- and long-acting benzodiazepines (compared with short-acting alone)), were associated with prolonged use, even as the number of prior episodes of benzodiazepine use increased, suggesting prescribers should keep these prescribing characteristics in mind, even in individuals who have a history of benzodiazepine use.

In previous studies, other factors associated with prolonged benzodiazepine use include older age, multi-morbidity, psychiatric co-morbidity, low-income, initial prescription by a psychiatrist, and prescription on discharge from hospital [5,14,21,39,40]. Future investigations should determine whether some of these sociodemographic and diagnostic factors interact with initial prescription characteristics to determine length of use. Future studies could also examine whether these results are generalizable to other sub-populations at higher risk of harms related to benzodiazepine use including individuals with cognitive impairment, or a history of falls.

This was a large population-based study in Canada’s most populous province, capturing all outpatient prescriptions for benzodiazepines across the province, regardless of payer, and linked administrative data reflecting all health service utilization in a publicly-funded system which supports the generalizability of our findings. There are, however, several limitations. Most importantly, our study relies on pharmacy dispensing records and assumes that individuals took medications as prescribed. It is possible that medications were stock-piled or not used as prescribed, which could result in misclassification of exposure variables and outcomes. Similarly, since z-drugs are not all captured in the NMS database, we were unable to include them in the analysis, and this may have resulted in misclassification of the exposure and outcome variables. However, the consistency between our primary analysis and the sensitivity analyses are reassuring. Other psychiatric medications such as SSRIs are also not captured in the NMS and therefore could not be included as covariates in the model. We were, however, able to include psychiatric diagnoses. Readers should note that we have presented hazard ratios (a commonly used statistic in time-to-event analyses) and that the results represent relative rate reductions rather than relative risk reductions [41]. Although we attempted to control for important confounders, there may be residual confounding by indication. Finally, as with all observational studies, our study is subject to bias from unmeasured confounding. Disease severity, use of non-prescribed benzodiazepines and social stability, for example, are not captured in administrative health data.

In this large population-based cohort study, initial benzodiazepine prescribing patterns were associated with time to benzodiazepine discontinuation. Prescribing shorter courses of benzodiazepines, use of a single benzodiazepine, use of a short-acting agent were associated with reduced likelihood of long-term benzodiazepine use. These findings suggest that simple changes to prescribing practice including limiting initial prescriptions to <7 days and choosing a single, short-acting agent could reduce prolonged benzodiazepine use and associated morbidity and mortality.

## Supporting information

S1 Appendix Supplementary Information. (DOCX)

S1 Data Dataset creation plan used for study planning. (DOCX)

S1 STROBE Checklist Completed STROBE checklist. *STrengthening the Reporting of OBservational studies in Epidemiology (STROBE) Statement – checklist of items that should be included in reports of observational studies, licensed under CC BY 4.0. von Elm E, Altman DG, Egger M, Pocock SJ, Gøtzsche PC, Vandenbroucke JP; STROBE Initiative. The Strengthening the Reporting of Observational Studies in Epidemiology (STROBE)statement: guidelines for reporting observational studies. PLoS Med. 2007 Oct 16;4 (10):e296. PMID: 17941714*.
(DOCX)

## Abbreviations

ACG: Adjusted Clinical Groups
ADGs: Aggregated Diagnosis Groups
aHR: adjusted Hazard Ratio
CI: Confidence Interval
DAD: Discharge Abstract Database
DMEs: Diazepam Milligram Equivalents
IQR: Interquartile Range
NACRS: National Ambulatory Care Reporting System
NMS: Narcotic Monitoring System
OAT: opioid agonist therapy
OHIP: Ontario Health Insurance Plan
RPDB: Registered Persons Database
STROBE: Strengthening the Reporting of Observational Studies in Epidemiology
