# Supplementary material for: Association between initial benzodiazepine prescribing patterns and time to benzodiazepine discontinuation: A population-based retrospective cohort study
Source: PLoS Med. 2026 Jun 18;23(6):e1005126. doi: 10.1371/journal.pmed.1005126 (PMC13278425; doi:10.1371/journal.pmed.1005126)
Supplement: S1 Appendix — (DOCX) [file pmed.1005126.s001.docx]

S1 Appendix

Table of Contents:

[Table A: Descriptions of all linked administrative databases used in the study 1](#_Toc230602009)

[Table B: Categorization of benzodiazepines as long-acting or short-acting based on half life 2](#_Toc230602010)

[Table C: DME conversion factors 3](#_Toc230602011)

[Table D: Definitions of covariates used 4](#_Toc230602012)

[Table E: Definitions of baseline variables 6](#_Toc230602013)

[Table F: Multivariable Cox Proportional hazards model of prescription factors associated with time to benzodiazepine discontinuation stratified by year of initial prescription 10](#_Toc230602014)

[Table G: Multivariable Cox Proportional hazards model of prescription factors associated with time to benzodiazepine discontinuation for first benzodiazepine episode during accrual period 2013-2020 11](#_Toc230602015)

[Figs A-D: Log-log plots 12](#_Toc230602016)

[References 16](#_Toc230602017)

## Table A: Descriptions of all linked administrative databases used in the study

| **Database** | **Description** |
| --- | --- |
| **Narcotics Monitoring System (NMS)** | The Narcotic Monitoring System is a mandatory prescription reporting system in Ontario, which is complete as of July 1, 2012. It captures all outpatient prescriptions for controlled substances in Ontario regardless of payment method. |
| **Registered Persons Database (RPDB)** | The RPDB provides demographic information and vital statistics on all persons with an Ontario health card (i.e. OHIP number). We used the RPDB to assess sex, age, and postal code of each individual’s primary residence. |
| **Drug-Alcohol Related Death Database** | DDARD is a dataset of confirmed cause of death from the Office of the Chief Coroner of Ontario and allows the accurate identification of the cause of death. In accordance with Ontario’s Coroners Act, all deaths that are sudden and unexpected, or unnatural, must be reported to the Coroner’s Office. |
| **Canadian Institute for Health Information (CIHI) Discharge Abstract Database (DAD)** | Captures details on diagnoses and procedures for all inpatient hospital stays in Ontario. This dataset has been validated through a re-abstraction study which found that the re-abstractor agreed at least in part with the original coder regarding the most responsible diagnosis approximately 85% of the time(1). |
| **CIHI National Ambulatory Care Reporting System (NACRS)** | Captures details on diagnoses and procedures for all emergency department visits in Ontario. |
| **CIHI Ontario Mental Health Reporting System (OMHRS)** | The OMHRS database contains demographic, administrative and clinical information for all adult admissions to designated inpatient mental health beds in Ontario. This includes beds in general hospitals as well as in specialty psychiatric facilities. We used OMHRS records to identify psychiatric admissions for the purpose of understanding psychiatric co-morbidity. |
| **Ontario Health Insurance Plan (OHIP) Database** | This database reflects physician claims through the Ontario Health Insurance Plan. Elements in this dataset include the patient and physician identifiers, the code for service provided, date of service, and associated diagnosis. We used OHIP claims to understand physician specialty, assess healthcare utilization, and in the definition of alcohol use disorder and insomnia. |
| **Chronic Obstructive Pulmonary Disease (COPD) Validated Database** | This database uses a validated definition to identify diagnosis dates for all people with COPD in Ontario (85.0% sensitivity, 78.4% specificity). (2) |
| **HIV Validated Database** | This database uses a validated definition to identify diagnosis dates for all people with HIV in Ontario (96.2% sensitivity, 99.6% specificity).(3) |
| **Ontario Diabetes Database** | This database uses a validated definition to identify diagnosis dates fo all people with diabetes in Ontario (90.0% sensitivity, 97.7% specificity).(4) |
| **ICES Physician Database (IPDB)** | IPDB is a dataset that contains yearly information about all physicians in Ontario including demographics, specialty, location and physician activity (billings, workload, types of services provided). For the purpose of our study, IPDB was used to ascertain provider specialty. |

## Table B: Categorization of benzodiazepines as long-acting or short-acting based on half life

| **Long-acting (~ > 24 hours)** | **Half life _(incl, metabolites)_** |
| --- | --- |
| Chlordiazepoxide | 100 |
| Clorazepate | 100 |
| Diazepam | 100 |
| Flurazepam | 100 |
| Clonazepam | 20-60  * 30-40 hours per product monograph |
| Nitrazepam | 16-55  *18-57 hours per product monograph, ~40 hours elderly |
| **Short-acting (~ < 24 hours)** |  |
| Alprazolam | 12-15 |
| Bromazepam | 8-30  *approx. 20 hours per product monograph |
| Lorazepam | 10-20 |
| Oxazepam | 5-25 |
| Temazepam | 10-20 |
| Triazolam | 1.5-5 |

## Table C: DME conversion factors

| **DRUGNAME** | **Equivalence to 10 mg diazepam (used in primary analysis)*** | **Conversion factor 1**  **(used in primary analysis)*** | **Equivalence to 10 mg diazepam (used in sensitivity analysis) ^$^** | **Conversion factor 2 (used in sensitivity analysis)^$^** |
| --- | --- | --- | --- | --- |
| **ALPRAZOLAM** | **0.5 mg** | **20** | **1 mg** | **10** |
| **BROMAZEPAM** | **5 mg** | **2** | **5 mg** | **2** |
| **CHLORDIAZEPOXIDE HCL** | **25 mg** | **0.4** | **25 mg** | **0.4** |
| **CHLORDIAZEPOXIDE HCL & CLIDINIUM BROMIDE** | **25 mg** | **0.4** | **25 mg** | **0.4** |
| **CLONAZEPAM** | **0.5 mg** | **20** | **1 mg** | **10** |
| **CLORAZEPATE DIPOTASSIUM** | **15 mg** | **0.67** | **15 mg** | **0.67** |
| **DIAZEPAM** | **10 mg** | **1** | **10 mg** | **1** |
| **FLURAZEPAM HYDROCHLORIDE** | **30 mg** | **0.33** | **30 mg** | **0.33** |
| **LORAZEPAM** | **1 mg** | **10** | **2 mg** | **5** |
| **NITRAZEPAM** | **10 mg** | **1** | **10 mg** | **1** |
| **OXAZEPAM** | **20 mg** | **0.5** | **30 mg** | **0.33** |
| **TEMAZEPAM** | **20 mg** | **0.5** | **20 mg** | **0.5** |
| **TRIAZOLAM** | **0.5 mg** | **20** | **0.5 mg** | **20** |

^*^Conversion factor 1 adapted from Brandt J, Alessi-Severini S, Singer A, Leong C. Novel Measures of Benzodiazepine and Z-Drug Utilisation Trends in a Canadian Provincial Adult Population (2001-2016). *J Popul Ther Clin Pharmacol*. Jan 22 2019;26(1):e22-e38. doi:10.22374/1710-6222.26.1.3(5)

^$^Conversion factor 2 adapted from Borrelli EP, Bratberg J, Hallowell BD, Greaney ML, Kogut SJ. Application of a diazepam milligram equivalency algorithm to assess benzodiazepine dose intensity in Rhode Island in 2018. *J Manag Care Spec Pharm*. Jan 2022;28(1):58-68. doi:10.18553/jmcp.2022.28.1.58(6)

## Table D: Definitions of covariates used

| **Variable** | **Database** | **Definition/Citation** |
| --- | --- | --- |
| Age | RPDB | Age at index using birth date in RPDB |
| Sex | RPDB | Sex as recorded in RPDB |
| Rurality of residence | RPDB | Rurality of residence using person’s postal code |
| Neighborhood income quintile | RPDB | Neighborhood income quintile using person’s postal code |
| Alcohol Use Disorder(7) | DAD, NACRS, OHIP | Physician or hospital related visit for alcohol use disorder using codes below (primary diagnosis) in the 2 years prior to index date:  **OHIP Codes:**  291, 303  **ICD-10 Codes:**  F10, K70, G312, G621, G721, I426, K292, K701, K704, K709, K860, Z502, Z714, Z8640 |
| Harmful sedative-hypnotic use or dependence | DAD, NACRS | Hospital visit for harmful sedative-hypnotic use or dependence using codes below (primary diagnosis) in the 2 years prior to index date:  **ICD-10 Codes:**  F131,F132, T423,T424d, T426,T427 |
| Insomnia(8) | OHIP, OMHRS, NACRS, DAD | Physician or hospital related visit for insomnia using codes below in the 2 years prior to index date:  **ICD-10 Codes:**  G47.0, F51.0, F51.4, F51.3, F51.5, F51.9, F51.2, F51.8, G47.2, G47.9, G47.8 DSM-5: 307.42, 327.01, 327.02, 780.52, 327.30, 327.31, 327.35, 327.36, 327.44, 327.80, 307.46, 307.45, 307.47, 292.85  **Ontario Mental Health Reporting System Provisional Diagnosis**: 12  **OHIP Code:** 307 |
| Psychotic disorders(9) | OHIP | Outpatient visit for psychotic disorders in the 1 year prior to and including index date:  **OHIP Codes**: 295,297,298 |
| Mood and anxiety disorders(9) | OHIP | Outpatient visit for mood and anxiety disorders in the 1 year prior to and including index date:  **OHIP Codes**: 296,300, 311 |
| John’s Hopkins Aggregated Diagnosis Groups (ADGs) (10) | OHIP, DAD | Patients were assigned a weighted score based on presence or absence of 32 ACG System Aggregated Diagnosis Groups (ADGs) characterizing medical conditions based on their use of inpatient and outpatient health care services in the preceding 2 years(10). ADGs were summed and grouped into quintiles. |
| Discipline of prescriber at initiation | IPDB, OHIP | Define pain prescriber as:  1)Psychiatry, where mainspecialty=PSYCHIATRY, CHILD & ADOLESCENT PSYCHIATRY, FORENSIC PSYCHIATRY, GERIATRIC PSYCHIATRY  2) Family medicine, where mainspecialty=GP/FP  3) Neurology=NEUROLOGY, PEDIATRIC NEUROLOGY  4) Emergency Medicine  5) Obstetrics and Gynecology  6) Internal Medicine where mainspecialty=INTERNAL MEDICINE, CARDIOLOGY, CLINICAL IMMUNOLOGY, ENDOCRINOLOGY, GASTROENTEROLOGY, GERIATRIC MEDICINE, HEMATOLOGY, NEPHROLOGY, RESPIROLOGY, RHEUMATOLOGY  3)Other, where mainspecialty = any other specialty  4) Missing, where mainspecialty = missing |

***See Table A for database definitions**

## Table E: Definitions of baseline variables

| **Variable** | **Database** | **Definition/Citation** |
| --- | --- | --- |
| Age | RPDB | Age at index using birth date in RPDB |
| Sex | RPDB | Sex as recorded in RPDB |
| Rurality of residence | RPDB | Rurality of residence using person’s postal code |
| Neighborhood income quintile | RPDB | Neighborhood income quintile using person’s postal code |
| Residing in long term care | CCRS, ODB, OHIP | Use the ODB+OHIP+CCRS to identify individuals in long term care in the 90 days prior to and including the index date |
| Concurrent opioid prescription | NMS | To identify prescriptions for opioids, use the master druglist to identify drug identification numbers (dins) where NMS_Group= “OPIOID” and OpioidMaintenanceTherapy= “N” and dclass ne ‘EXCL’  Pull all non OAT opioid prescriptions in the 30 days prior to and including index date. Check if the opioid prescription servdate+ days supplied overlaps benzodiazepine index date. If it does overlap index date, ‘Yes’, concurrent opioid prescription. |
| Alcohol Use Disorder(7) | DAD, NACRS, OHIP | Physician or hospital related visit for alcohol use disorder using codes below (primary diagnosis) in the 2 years prior to index date:  **OHIP Codes:**  291, 303  **ICD-10 Codes:**  F10, K70, G312, G621, G721, I426, K292, K701, K704, K709, K860, Z502, Z714, Z8640 |
| Stimulant harmful use or dependence | DAD, NACRS | Hospital visit for harmful stimulant use or dependence using codes below (primary diagnosis) in the 2 years prior to index date:  **ICD-10 codes:**  F140- F159, T436, T405, |
| Harmful sedative-hypnotic use or dependence | DAD, NACRS | Hospital visit for harmful sedative-hypnotic use or dependence using codes below (primary diagnosis) in the 2 years prior to index date:  **ICD-10 Codes:**  F131,F132, T423,T424d, T426,T427 |
| Other medications prescribed in the last 6 months | NMS | Other medications prescribed in the 180 days prior (excluding index) to cohort entry. Use the NMS master druglist.   - - Stimulants (n, %)     - NMS_group = “STIM”   - Non-OAT opioid (n, %)   NMS_Group= “OPIOID” and OMT= “N” and dclass ne ‘EXCL’   - - OAT opioid (n, %)   NMS_Group= “OPIOID” and OMT= “Y”   - - Barbiturates (n, %)     - NMS_group = “BARB”   - THC products (n, %)     - NMS_group= “THC” |
| Psychotic disorders(9) | OHIP | Outpatient visit for psychotic disorders in the 1 year prior to and including index date:  **OHIP Codes**: 295,297,298 |
| Mood and anxiety disorders(9) | OHIP | Outpatient visit for mood and anxiety disorders in the 1 year prior to and including index date:  **OHIP Codes**: 296,300, 311 |
| Substance use (9) | OHIP | Outpatient visit for substance use disorders in the 1 year prior to and including index date:  **OHIP Codes:** 291, 292, 303,304 |
| Behavioural and neurodevelopmental disorders | OHIP | Outpatient visit for behavioural and neurodevelopmental disorders in the 1 year prior to and including index date:  OHIP Codes: 299, 313, 314, 315 |
| ED visit or hospitalization for any mental health or addictions | NACRS, DAD, OMHRS | **ICD-10-CM Codes (OMHRS DSM5)**  ·   ICD10CMCODE_DISCH1 = Any OMHRS (includes missing, except for neurocognitive disorders in primary diagnosis):    F01.50 without behavioural disturbances  F01.51 with behavioural disturbances  F02.80 without behavioural disturbances  F02.81 with behavioural disturbances  F05 Delirium due to multiple etiologies  G31.84 Mild neurocognitive disorder due to ^  G31.9 Probable major neurocognitive disorder due to ^, possible major neurocognitive disorder due to ^  R41.0 Other specified delirium and unspecified delirium  R41.9 Unspecified neurocognitive disorder    Where, ^ is any of the following:  Alzheimer’s disease  Frontotemporal lobular degeneration  Lewy bodies  Vascular neurocognitive disorder  Traumatic brain injury <  HIV infection  Prion disease  Parkinson’s disease  Huntington’s disease  Another medical condition  Multiple etiologies   - Exclude if ICD10CMCODE_DISCH1 missing and PROVDX_DSM5CODE_ADM1 = 17     **ICD-10-CA Codes (DAD/NACRS)**  DX10CODE1= F06-F99  or DX10CODE2-DX10CODE10 = X60-X84, Y10-Y19, Y28 when DX10CODE1 ne F06-F99 |
| HIV | HIV2020 | HIV diagnosis date<Index date using validated cohort |
| COPD | COPD | COPD diagnosis date<index date using validated cohort |
| Diabetes | Diabetes | Diabetes diagnosis date<index date using validated cohort |
| Insomnia(8) | OHIP, OMHRS, NACRS, DAD | Physician or hospital related visit for insomnia using codes below in the 2 years prior to index date:  **ICD-10 Codes:**  G47.0, F51.0, F51.4, F51.3, F51.5, F51.9, F51.2, F51.8, G47.2, G47.9, G47.8 DSM-5: 307.42, 327.01, 327.02, 780.52, 327.30, 327.31, 327.35, 327.36, 327.44, 327.80, 307.46, 307.45, 307.47, 292.85  **OMHRS Code**: 12  **OHIP Code:** 307 |
| Discipline of prescriber at initiation | IPDB, OHIP | Define pain prescriber as:  1)Psychiatry, where mainspecialty=PSYCHIATRY, CHILD & ADOLESCENT PSYCHIATRY, FORENSIC PSYCHIATRY, GERIATRIC PSYCHIATRY  2) Family medicine, where mainspecialty=GP/FP  3) Neurology=NEUROLOGY, PEDIATRIC NEUROLOGY  4) Emergency Medicine  5) Obstetrics and Gynecology  6) Internal Medicine where mainspecialty=INTERNAL MEDICINE, CARDIOLOGY, CLINICAL IMMUNOLOGY, ENDOCRINOLOGY, GASTROENTEROLOGY, GERIATRIC MEDICINE, HEMATOLOGY, NEPHROLOGY, RESPIROLOGY, RHEUMATOLOGY  3)Other, where mainspecialty = any other specialty  4) Missing, where mainspecialty = missing |
| Number of physician visits in 1 year prior to index date | OHIP | Use %getohip (source=nonlab, spec=physician, restrict to visits with an office location: location=O, H, L, or P) Count one claim per person per physician per day. |
| Number of ED visits in 1 year prior to index date | NACRS | Count the number of unique ED visits. |
| Number of hospitalizations in 1 year prior to index date | DAD | Count the number of unique hospital episodes. |
| John’s Hopkins Aggregated Diagnosis Groups (ADGs) (10) | OHIP, DAD | Patients were assigned a weighted score based on presence or absence of 32 ACG System Aggregated Diagnosis Groups (ADGs) characterizing medical conditions based on their use of inpatient and outpatient health care services in the preceding 2 years(10). ADGs were summed and grouped into quintiles. |

## Table F: Multivariable Cox Proportional hazards model of prescription factors associated with time to benzodiazepine discontinuation stratified by year of initial prescription

|  | **Adjusted Hazard Ratio (95% CI)** | | | |
| --- | --- | --- | --- | --- |
|  | **Year of initial prescription 2013-2016** | | **Year of initial prescription 2017-2020** | |
| **Days supplied at index** |  |  |  | |
| <7 days | 1.00 | | 1.00 | |
| 8-14 days | 0.58 (0.57, 0.58) | | 0.50 (0.50, 0.50) | |
| 15-30 days | 0.27 (0.27-0.28) | | 0.24 (0.24, 0.24) | |
| >30 days | 0.15 (0.15, 0.15) | | 0.13 (0.13, 0.13) | |
| **Type of benzodiazepine dispensed at index** | | | |  |
| Short-acting | 1.00 | | 1.00 | |
| Short- and long-acting | 0.86 (0.81, 0.91) | | 0.80 (0.76, 0.87) | |
| Long-acting | 0.79 (0.79, 0.80) | | 0.80 (0.80, 0.81) | |
| **Number of benzodiazepines dispensed at index** | | | |  |
| 1 | 1.00 | | 1.00 | |
| > 2 | 0.56 (0.54, 0.59) | | 0.65 (0.60, 0.69) | |
| **Mean daily dose of index prescription in Diazepam Milligram Equivalents** | | | | |
| <5 | 1.00 | | 1.00 | |
| >5 - <10 | 1.03 (1.02, 1.03) | | 1.04 (1.03, 1.05) | |
| >10-<20 | 1.05 (1.05, 1.06) | | 1.02 (1.01, 1.02) | |
| >20 | 1.01 (1.01, 1.02) | | 0.95 (0.94, 0.96) | |

Models were adjusted for age, rurality of residence, sex, income quintile, alcohol use disorder, harmful sedative-hypnotic use or dependence, receipt of Opioid Agonist Therapy in the last 6 months, psychotic disorders, anxiety and mood disorders, insomnia, discipline of prescriber, and Aggregated Diagnosis Groups.

## Table G: Multivariable Cox Proportional hazards model of prescription factors associated with time to benzodiazepine discontinuation for first benzodiazepine episode during accrual period 2013-2020

|  | **Adjusted Hazard Ratio**  **(95% Confidence Interval)** | | |
| --- | --- | --- | --- |
| **Days supplied at index** |  |  |  |
| <7 days | 1.00 | | |
| 8-14 days | 0.55 (0.55, 0.55) | | |
| 15-30 days | 0.26 (0.26, 0.26) | | |
| >30 days | 0.14 (0.14, 0.14) | | |
| **Type of benzodiazepine dispensed at index** | | | |
| Short-acting | 1.00 | | |
| Short- and long-acting | 0.89 (0.85, 0.93) | | |
| Long-acting | 0.82 (0.82, 0.83) | | |
| **Number of benzodiazepines dispensed at index** | | | |
| 1 | 1.00 | | |
| > 2 | 0.60 (0.57, 0.62) | | |
| **Mean daily dose of index prescription in Diazepam Milligram Equivalents** | | | |
| <5 | 1.00 | | |
| >5 - <10 | 1.04 (1.04, 1.05) | | |
| >10-<20 | 1.06 (1.05, 1.06) | | |
| >20 | 1.00 (1.00, 1.01) | | |

Models were adjusted for age, rurality of residence, sex, income quintile, alcohol use disorder, harmful sedative-hypnotic use or dependence, receipt of Opioid Agonist Therapy in the last 6 months, psychotic disorders, anxiety and mood disorders, insomnia, discipline of prescriber, and Aggregated Diagnosis Groups.

## Figs A-D: Log-log plots

**Fig A: Log-log plot for days supplied**

**
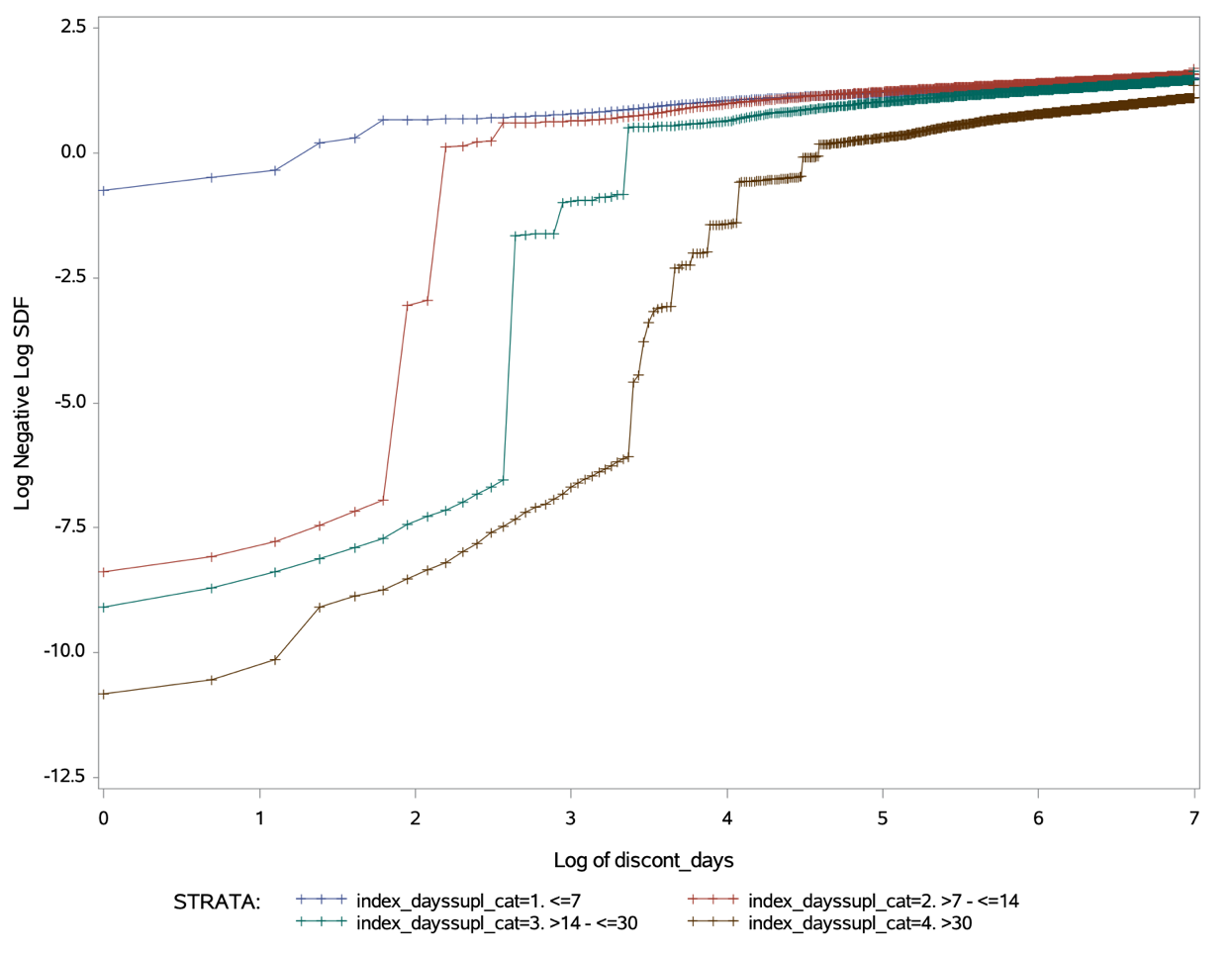
**

**Fig B: Log-log plot for number of benzodiazepines prescribed**

**
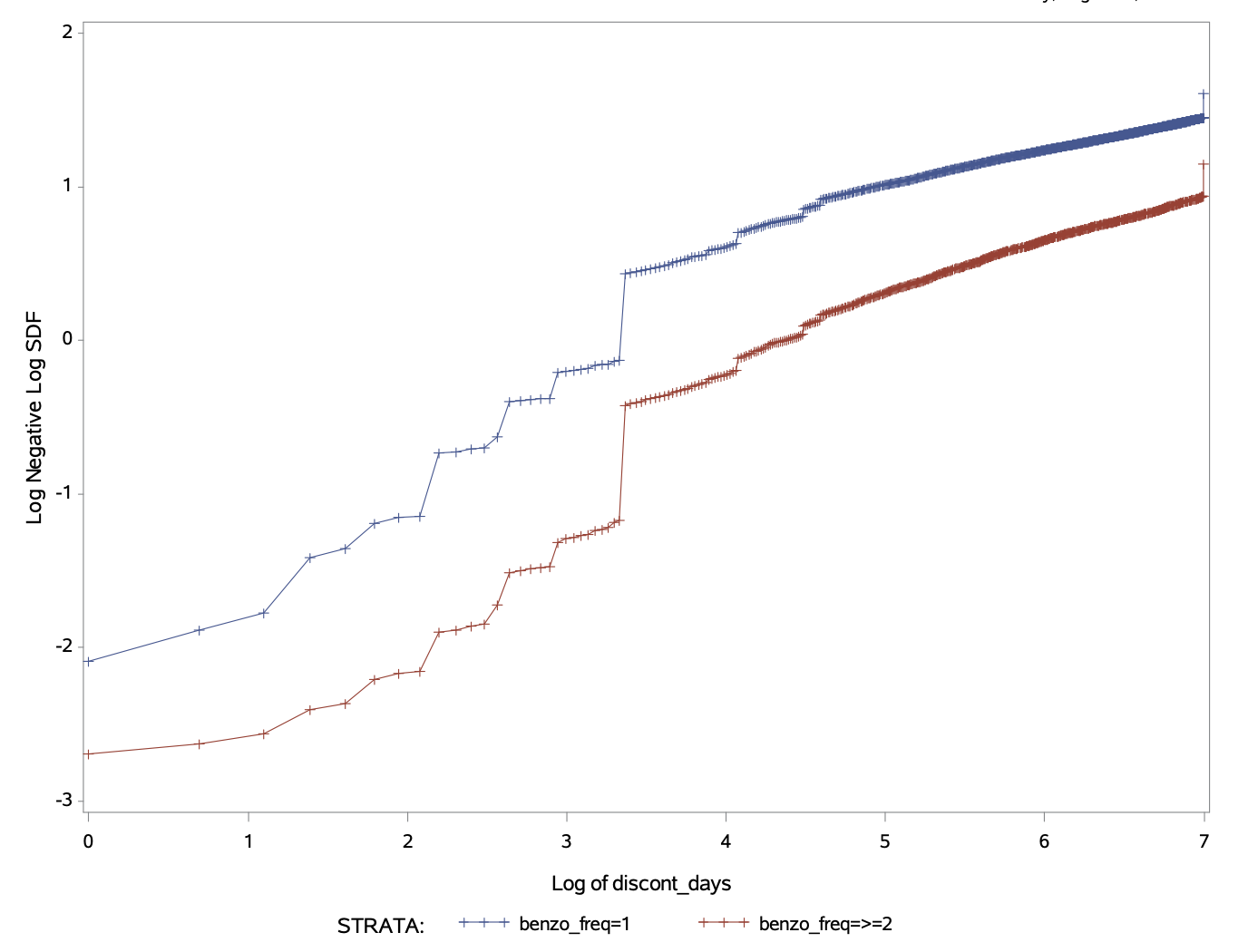
**

**Fig C: Log-log plot for benzodiazepine type**

**
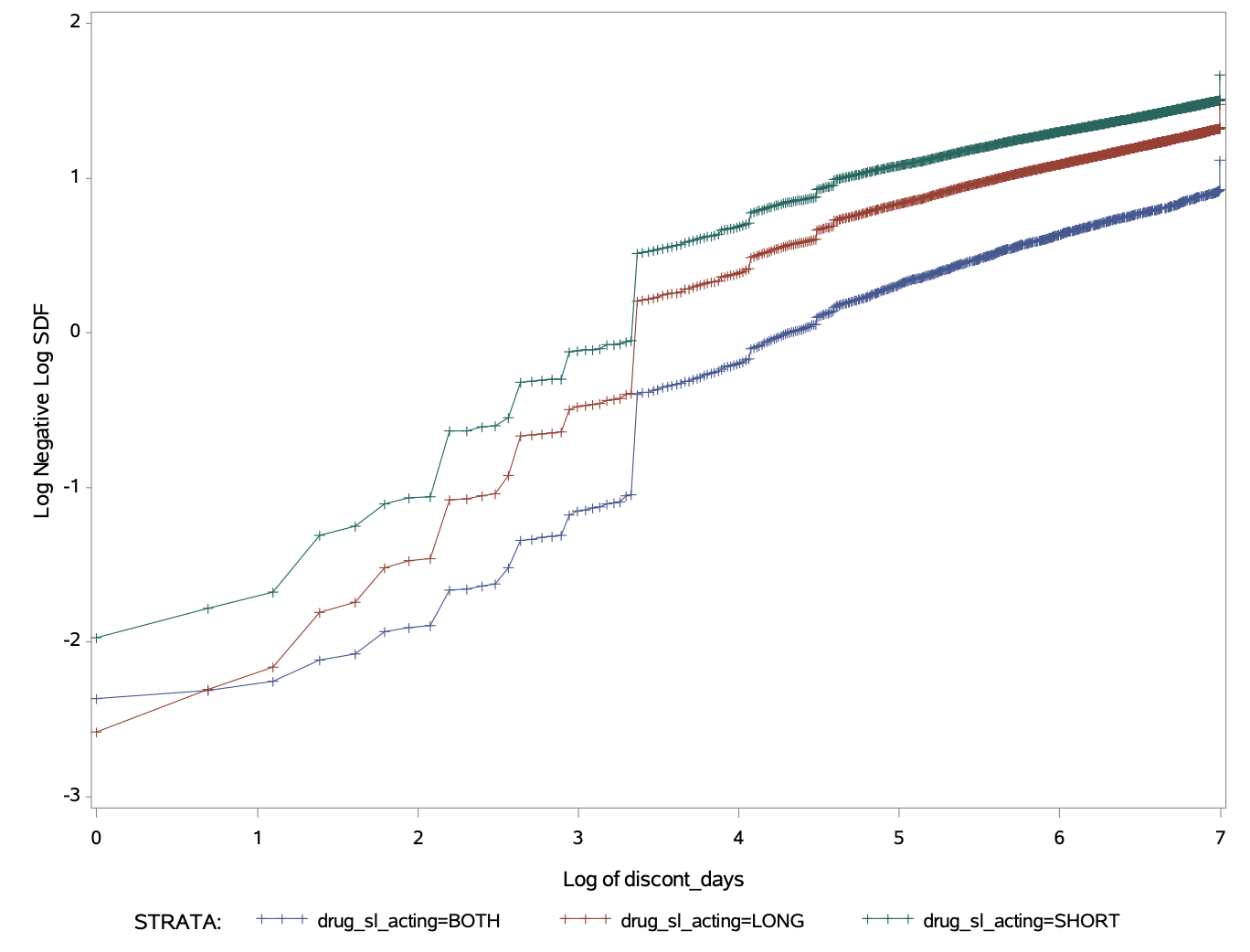
**

**Fig D: Log-log plot for Diazepam Milligram Equivalents**

**
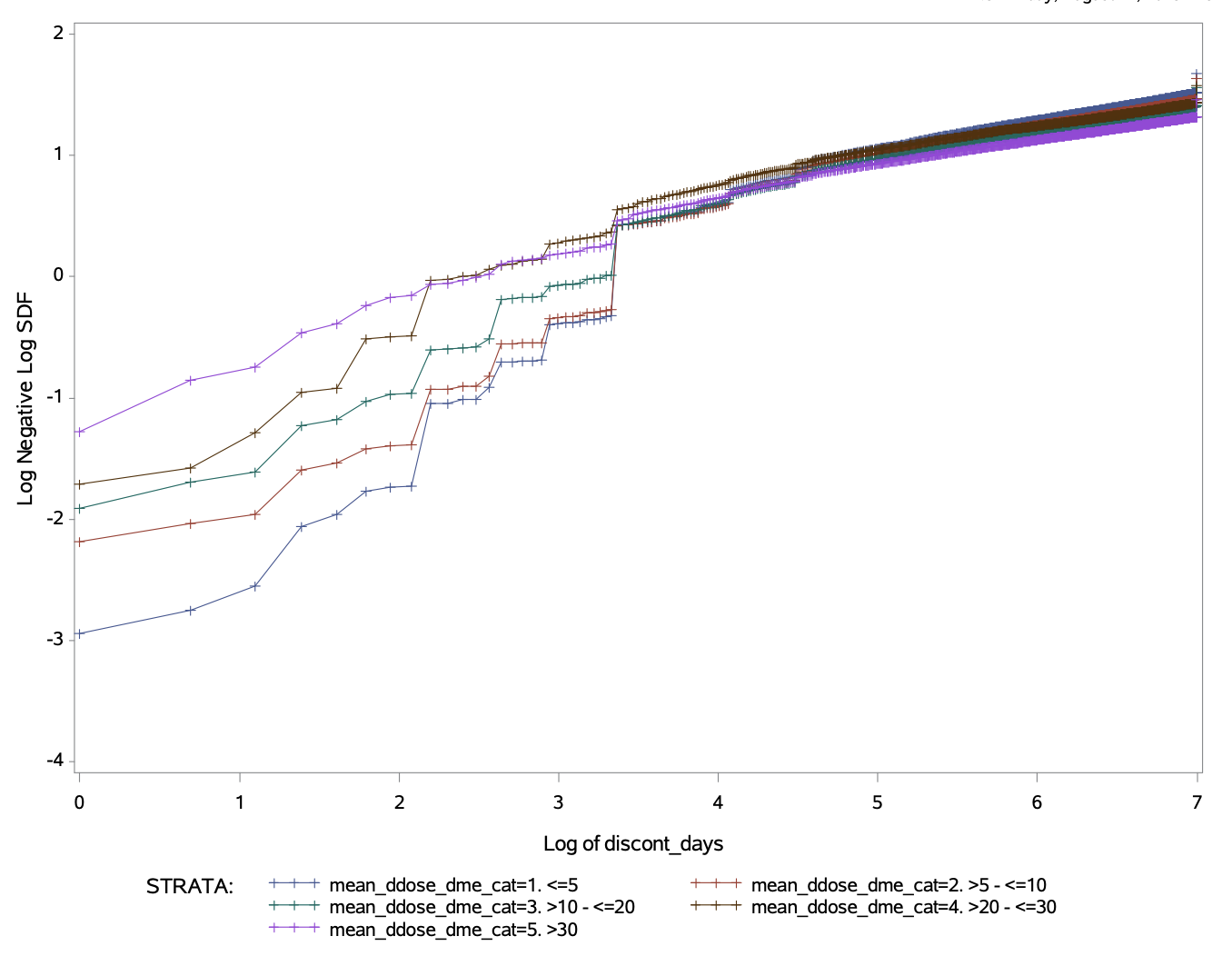
**

## References

1. Juurlink D, Preyra C, Croxford R, Chong A, Austin P, Tu J, et al. Canadian Institute for Health Information Discharge Abstract Database: A Validation Study.  . Toronto; 2006.

2. Gershon AS, Wang C, Guan J, Vasilevska-Ristovska J, Cicutto L, To T. Identifying individuals with physcian diagnosed COPD in health administrative databases. COPD. 6. England2009. p. 388-94.

3. Antoniou T, Zagorski B, Loutfy MR, Strike C, Glazier RH. Validation of case-finding algorithms derived from administrative data for identifying adults living with human immunodeficiency virus infection. PLoS One. 2011;6(6):e21748.

4. Lipscombe LL, Hwee J, Webster L, Shah BR, Booth GL, Tu K. Identifying diabetes cases from administrative data: a population-based validation study. BMC Health Serv Res. 2018;18(1):316.

5. Brandt J, Alessi-Severini S, Singer A, Leong C. Novel Measures of Benzodiazepine and Z-Drug Utilisation Trends in a Canadian Provincial Adult Population (2001-2016). J Popul Ther Clin Pharmacol. 2019;26(1):e22-e38.

6. Borrelli EP, Bratberg J, Hallowell BD, Greaney ML, Kogut SJ. Application of a diazepam milligram equivalency algorithm to assess benzodiazepine dose intensity in Rhode Island in 2018. J Manag Care Spec Pharm. 2022;28(1):58-68.

7. Nickel NC, Bolton J, MacWilliam L, Ekuma O, Prior H, Valdivia J, et al. Health and Social Outcomes Associated with High-Risk Alcohol Use. Winnipeg, MB; 2018.

8. Thorpy MJ. Classification of sleep disorders. Neurotherapeutics. 2012;9(4):687-701.

9. Team MR. The Mental Health of Children and Youth in Ontario; 2017 Scorecard. Technical Appendix. Toronto, ON: Institute for Clinical Evaluative Sciences; 2017.

10. The John Hopkins University Bloomberg School of Public Health HSRDC. The John Hopkins ACG Case-Mix System Version 6.0 Release Notes. Weiner EiCJP, editor: The John Hopkins University  2003.
