# Supplementary material for: Association between initial benzodiazepine prescribing patterns and time to benzodiazepine discontinuation: A population-based retrospective cohort study
Source: PLoS Med. 2026 Jun 18;23(6):e1005126. doi: 10.1371/journal.pmed.1005126 (PMC13278425; doi:10.1371/journal.pmed.1005126)
Supplement: S1 Data — (DOCX) [file pmed.1005126.s002.docx]

| Note this Dataset Creation Plan encompases two objectives. For the purpose of the PLOS Medicine paper, please see Objective 2 in the below document. | | | | | | |
| --- | --- | --- | --- | --- | --- | --- |
| **Project Title:** | Prescribing characteristics associated with successful benzodiazepine taper | | | | | |
| **Project TRIM number:** | 2023 0990 660 000 | | | | | |
| **Research Program:** | CDP | | | | | |
| **Site:** | ICES Central | | | | | |
| **Project Purpose and Objectives:** | *Insert Project Purpose and Objectives as listed in the approved ICES Project PIA* | | | | | |
|  | 1.To describe time to discontinuation of benzodiazepines following treatment initiation in women compared with men 18 years or older in Ontario, and 2. To understand patterns of initial prescription by sex and their relationship with time to discontinuation. | | | | | |
| **ICES Project PIA Initial Approval Date:** | *The ICES Employee or agent who is responsible for creating the Project Dataset(s) is responsible for ensuring there is an approved ICES Project PIA and verifying the date of approval prior to creating the Project Dataset(s)* | | | | | |
|  | 2022Jul-17 | | | | | |
| **Principal Investigator (PI):** | Dr. Nikki Bozinoff | | | | | |
| **Check the applicable box if the PI is an ICES Student/Trainee** | ICES Student  ICES Fellow  ICES Post-Doctoral Trainee  Visiting Scholar | | | | | |
| **Responsible ICES Scientist:** | *Name the Responsible ICES Scientist if the PI is not a Full Status ICES Scientist* | | | | | |
|  | Dr. Tara Gomes | | | | | |
| **Project Team Member(s) Responsible for Project Dataset Creation and/or Statistical Analysis and date joined (list all):** | *All person(s) (ICES Analyst, Appointed Analyst, Analytic Epidemiologist, PI, and/or Student) responsible for creating the Project Dataset(s) and/or statistical analysis on the Research Analytics Environment (RAE) and/or Data Safe Haven (DSH) and the date they joined the project must be recorded* | | | | | |
|  |  | | | yyyy-mon-dd | | |
| **Project Team Member(s) who will request RAE and/or DSH folder access (list all):** | *List the project team member responsible for dataset creation who will request access for all members requiring RAE and/or DSH project folder access (e.g. analyst, methodologist, student, etc).* | | | | | |
|  | Dr. Tara Gomes, Divya Prasad | | 2022-Jul-28 | | |  |
| **Other ICES Project Team Members and date joined (list all):** | *All other Research Project Team Members (e.g., Research Administrative Assistants, Research Assistants, Project Managers, Epidemiologists) and the date they joined the project must be recorded* | | | | | |
|  | Dr. Simone Vigod  Maria Zhang (RPh)  Beth Sproule (RPh)  Dr. Tanya Hauck  Dr. Matthew Sloan  Dr. Jennifer Wyman  Divya Prasad  Name redacted to protect lived experience advisory member name at their request.  Dr. Robert Kleinman | | | 2021-Jul-28 | | |
| **Confirmation that DCP is consistent with Project Objectives:** | *The following individuals must confirm that the ICES Data provided for in this DCP is relevant (e.g., with respect to cohort, timeframe, and variables) and required to achieve the purpose(s) stated in the ICES Project PIA prior to initial Project Dataset creation: 1) PI; 2) Responsible ICES Scientist if the PI is not a Full Status ICES Scientist, or a second ICES Scientist or the Scientific Program Lead if the PI is creating both the DCP and the Project Dataset[s]; 3) ICES Research and Analysis Staff creating the DCP; and 4) ICES Analytic Staff (ICES Employee or agent responsible for creating the Project Dataset[s]). Documentation of this confirmation may be delegated either verbally or via e-mail.* | | | | | |
|  | ***Nikki Bozinoff*** |  | | | 2022-Sept-28 | |
|  | ***Tara Gomes*** |  | | 2022-SEP-27 | | |
|  | ***ICES Research and Analysis Staff Creating the DCP*** |  | | yyyy-mon-dd | | |
|  | ***ICES Analytic Staff Ayesha Asaf*** |  | | 2022-SEP-27 | | |
| **Designated ICES Research and Analysis Staff accountable for Project Documentation:** | *The person named (ICES staff) is accountable for ensuring that the approved ICES Project PIA, ICES Project PIA Amendments, and DCP are saved on the T Drive, ensuring ICES Project PIA Amendments are submitted as required, ensuring DCP Amendments are documented, and sharing the final DCP with the PI/Responsible ICES Scientist at project completion* | | | | | |
|  | Divya Prasad | | | | | |

| **DCP Creation Date and Author:** | *Date DCP was finalized prior to Project Dataset(s) creation* | *Name of person who created the DCP* |
| --- | --- | --- |
|  | ***Date*** | ***Dr. Nikki Bozinoff*** |
|  | yyyy-mon-dd |  |

| ICES DataThis Section must be Completed Prior to Project Dataset(s) Creation | |
| --- | --- |
| *The ICES Employee or agent who is responsible for creating the Project Dataset(s) must ensure that this list includes only data listed in the ICES Project PIA*  *Changes to this list after initial ICES Project PIA approval require an ICES Project PIA Amendment* | *Mandatory for all datasets that are available by individual year* |
| ***General Use Datasets – Health Services*** | ***Years (where applicable)*** |
| CIHI DAD | 2012 – 2021 |
| NACRS | 2012 – 2021 |
| OHIP | 2012 – 2021 |
| OMHRS | 2012 – 2021 |
| ODB | 2012 - 2021 |
| CCRS | 2012 - 2021 |
| ***General Use Datasets – Care Providers*** |  |
| IPDB | 2012 – 2021 |
| See list |  |
| ***General Use Datasets – Population*** |  |
| RPDB | 2012 – 2021 |
| CENSUS | 2012 – 2021 |
| ***General Use Datasets – Coding/Geography*** |  |
| DIN | 2012 – 2021 |
| PCCF | 2012 – 2021 |
| ***General Use Datasets - Facilities*** |  |
| See list |  |
| ***General Use Datasets - Other*** |  |
| HIV | 2012-2021 |
| COPD | 2012-2021 |
| ODD | 2012-2021 |
| ***Controlled Use Datasets*** |  |
| See list |  |
| See list |  |
| ***Other Datasets (including PSD and PDC data)*** |  |
| NMS | 2012 – 2021 |

| Project Amendments and Reconciliation | | | |
| --- | --- | --- | --- |
| **ICES Project PIA Amendment History (add additional rows as needed):** | *Privacy approval date* | *Person who submitted amendment* | *Note that any changes to the list of ICES Data or Project Objectives require an ICES Project PIA Amendment* |
|  | ***Date*** | ***Name*** | ***Amendment*** |
|  | 2022-Aug-24 | Samantha Singh and Divya Prasad | Two databases added: CCRS and ODB. |
| **DCP Amendment History (add additional rows as needed):** | *Date DCP amended* | *Person who made the DCP amendment* | *Note that any DCP amendments involving changes to the list of ICES Data or Project Objectives require an ICES Project PIA Amendment* |
|  | ***Date*** | ***Name*** | ***Amendment*** |
|  | 2023-03-31 | Nikki Bozinoff | Clarification that benzodiazepines with the same name should be considered a single benzodiazepine script |
|  | 2023-04-14 | Nikki Bozinoff | Added diagnostic codes for insomnia |
|  | 2023-04-14 | Nikki Bozinoff | Added outpatient mental health codes |
|  | 2023-06-16 | Nikki Bozinoff | Change mental health covariates in model to outpatient ones; initial prescription duration – make categorical variable; update analytic plan |
|  | 2023-08-03 | Nikki Bozinoff | Add insomnia to the model as a covariate. Remove stimulant use disorder, remove other mental health disorders, remove behavioural disorders, remove inpatient anxiety codes |
|  | 2023-12-11 | Nikki Bozinoff | Baseline table, add standardized differences |
|  | 2023-12-11 | Nikki Bozinoff | Update to prescriber variable definition |
|  | 2023-12-11 | Nikki Bozinoff | Update to mental health diagnoses based on hospitalization and ED visits. |
|  | 2023-12-11 | Nikki Bozinoff | Update to secondary exposure in model – group 1 and 2+ benzos at index. |
|  | 2023-12-11 | Nikki Bozinoff | Median time from KM curve |
|  | 2023-12-11 | Nikki Bozinoff | Update to descriptive information requested |
|  | 2023-12-11 | Nikki Bozinoff | Add secondary exposure mean daily dose. Sensitivity analysis added with different conversion factors. |
|  | 2024-03-09 | Nikki Bozinoff | Add categories to mean daily dose exposure |
|  | 2024-03-09 | Nikki Bozinoff | Add exclusion related to data quality |
| **Note primary analysis was conducted in mid March 2024** | | | |
|  | 2026-01-05 | Nikki Bozinoff | Provide Objective 2 model stratified by time period early vs. late (2013-2016 vs. 2017-2020) |
|  | 2026-01-05 | Nikki Bozinoff | Provide Objective 2 model using first episode in the accrual period. |
|  | 2026-01-05 | Nikki Bozinoff | Include all first and recurrent episodes as of July 1, 2014 allowing a 2 year look back. Run a PWP-TT model overall and stratified by episode number |
| **Date Programs/DCP reconciled** | *The person(s) creating the dataset and/or analyzing the data are responsible for ensuring that the final DCP reflects the final program(s) when the project is completed* | | |
|  | yyyy-mon-dd | | |

| Project Cohort | | |
| --- | --- | --- |
| **Study Design** | Cohort study  Matched cohort study  Case-control study  Cross-sectional study  Other (specify): | |
| **Index Event / Inclusion Criteria**  *(please ensure index event / inclusion criteria are specified with data sources, variables, study period and values or codes)* | Include persons 18 years or older with a new benzodiazepine treatment episode. A treatment episode is defined as continuous use of a benzodiazepine (exclude midazolam, clobazam) and no other benzodiazepine prescription in the 182 days prior to cohort entry (using NMS Cohort Multiple)  The cohort will be created using %nms_multiple macro and the following parameters::   - Accrual Period: 01JAN2013 to 31DEC2020 - End follow-up: 31DEC2021 - New use defined as no prior prescription for any benzodiazepine in previous 182 days - Drugs: use all DINs in attached druglist (Drug List 1) - Definition of ongoing use: lookforward 1.5x for next prescription. Use a minimum grace (mingrace) of 30 days. - Do not add together leftover tablets (addleftover=F) - End date = last servdate + days supply -1. If multiple prescriptions dispensed on end date, select prescription with longest days supply.   To identify prescriptions for benzodiazepines, use the ODPRN NMS master druglist to identify drug identification numbers (dins) where NMS_GROUP= ‘BZO’ and ORAL_TAB=Y. I   - - If prescriptions for two separate benzodiazepines are overlapping, assume that they are taken concurrently rather than sequentially. | |
| **Estimated Size of Cohort** |  | |
| **Exclusions** *(in order)  (common exclusions are listed in grey italics for consideration)* | *Step* | Description |
|  | 1 | 1. Individuals with missing/invalid IKN, 2. missing age or sex 3. Age<18 or age>105 4. death date prior to benzodiazepine initiation date (BEGIN date) 5. Not an Ontario resident (PRCDDABLK,1,2 ne ‘35’) 6. Individuals who received palliative care services in 6 months prior to cohort entry    1. Any CIHI-DAD hospitalization with patient service (PATSERV) = 58. (source=inpatient, acute care hospital=T) OR    2. Any OHIP billing with one of the following billing codes: OHIP FEECODE A945, B998, C945, C882, C982, K023, W872, W882, W972 or W982. 7. Exclude episodes with any clobazam or midazolam (use Drug List 2 for benzos to include) 8. Exclude episodes with a mean daily dose of index prescription >1000 DMEs based on conversion #1.   Note to analyst. Please report number of episodes excluded with each of these exclusions, as well as the total included at this point.  For the purpose of the analysis, please randomly choose one episode per individual to include. |

| Project Time Frame Definitions | | |
| --- | --- | --- |
| Look-back Window  Observation Window  (in which to look for outcomes)  **Index Event Date**  Accrual Window  Max Follow-up Date | |  |
| **Accrual Start/End Dates** | January 1, 2013- December 31, 2020 |  |
| **Max Follow-up Date** | Dec 31, 2021 |  |
| **When does observation window terminate?** | Outcomes measured at 3 years follow up from index, or maximum of Dec 31, 2021 |  |
| **Lookback Window(s)**  *(please ensure lookback windows are defined with start and end dates and in relation to the index event date)* | 182 days prior to index date (6 months) |  |

| Variable Definitions (add additional rows as needed) *A few key points to keep in mind:*   1. *Please ensure codes, data sources, diagnosis types and lookback periods (if applicable) are provided for all definitions listed below and that codes are provided in Excel format. If borrowing codes from another project, please list all the codes here* 2. *There are maximum number of digits that can be specified using ICES data (ICD 9 CA codes are up to maximum of 4 digits, ICD 10 CA codes are 6 digits, OHIP diagnosis codes are 3 digits)* | | |
| --- | --- | --- |
| **Main Exposure or Risk Factor** | Sex |  |
| **Secondary Exposure** | 1. Type of benzodiazepine(s) prescribed at index (name of drug(s) based on DIN (NMS)). Then categorized in three ways 1) short acting, long-acting or short AND long-acting (see Supplement 1), and 2) one benzo dispensed at baseline vs 2+. Note for the purpose of defining the number of benzodiazepines, please consider multiple prescriptions with the same drug name to be a single benzodiazepine. 2. Duration of index prescription in days. Note if more than one prescription occurred on index, choose the length of the one with the longest dayssuppl. Please categorize variable as follows:   < 7 days  >7- <14 days  >14- <30 days  >30 days   1. Mean daily dose of index prescription(s) in DMEs. Calculate the total Diazepam Milligram Equivalents (DMEs) for each prescription based on drug name and strength of benzodiazepine (benzodiazepine strength x Conversion Factor = DMEs (Supplement 1 ). Then, calculate the mean daily dose strength in DME*quantity dispensed/days supplied. If more than one prescription at index, sum mean daily dose.   Please categorize variables as follows:  < 5 DMEs (ref)  >5-<10 DMEs  >10-<20 DMEs  >20 |  |
| **Primary Outcome Definition** | - Time to discontinuation defined as episode END date. **Primary Censoring criteria:** Censor on death, 3 years from index, or maximum follow-up (Dec 31, 2021) |  |
| **Baseline Characteristics** | **Defined at benzo start date (BEGIN date), unless otherwise specified:**   1. Age – RPDB    - Median (IQR)    - Age group (n, %)      - 18-24      - 25-34      - 35-44      - 45-64      - 65+ 2. Sex (n, %) (RPDB) 3. Income quintile (n, %) (RPDB incquint variable)    - Q1, Q2, Q3, Q4, Q5, missing 4. Urban/rural location of residence (n, %) (RPDB rural variable)    - Rural, Urban, missing 5. LTC (n, %) (ODB, OHIP, CCRS_LTC) (Supplement 1) 6. Concurrent opioid prescription (n,%), defined as any opioid prescription that overlaps index date (Supplement 1) 7. Alcohol use disorder (n, %) (Supplement 1) in the 730 days prior (including index date) to cohort entry 8. Insomnia (n, %) (Supplemnent 1) in the 730 days prior (including index date) to cohort entry 9. Other medications prescribed in the 180 days prior (excluding index) to cohort entry. Use the ODPRN NMS master druglist:    - Stimulants (n, %)      - NMS_group = “STIM”    - Non-OAT opioid (n, %)   NMS_Group= “OPIOID” and OMT= “N” and dclass ne ‘EXCL’   - - OAT opioid (n, %)   NMS_Group= “OPIOID” and OMT= “Y”   - - Barbiturates (n, %)     - NMS_group = “BARB”   - THC products     - NMS_group= “THC”  1. Mental health diagnoses (Supplement 1) defined using any ED visit or hospitalization for mental health and addictions diagnoses in the 2 years prior (excluding index) to benzodiazepine initiation (n, %) 2. Mental health diagnoses (Psychotic disorders, anxiety and mooddisorders, substance use, Behavioural & Neuro-developmental disorders) based on outpatient visits (supplement 1) defined using outpatient visits in the 1 year prior to and including index (n, %) 3. COPD (n, %)­(COPD Cohort) anytime prior (including index date) to cohort entry 4. Diabetes (n, %)(Ontario Diabetes Dataset) any time prior (including index date) to cohort entry 5. HIV (n, %) (Ontario HIV Database) anytime prior (including index date) to cohort entry 6. Specialty of physician prescriber (n, %)(supplement 1): convert presc_i on index prescription to physnum, and use IPDB to determine mainspecialty as:   Psychiatry, Family medicine, Neurology, Emergency Medicine, Internal Medicine, Obstetrics and Gynecology, Other, missing.   1. Health system utilization in the 365 days prior (excluding index date) to cohort entry    - Number of physician visits (Median, IQR)   Use %getohip (source=nonlab, spec=physician, restrict to visits with an office location: location=O, H, L, or P) Count one claim per person per physician per day.   - - Number of ED visits (Median, IQR, N(%) with 1+ ED visit)     - Use %getnacrs (source=ed, dedup=T, inclfrom_typee=F, inclto_typeip=T , inclscheduled=F , inclnotseen=F)   - Number of hospitalizations (Median, IQR, N(%) with 1+ hospitalization)     - Use %getdadsds (source=inpatient, refdate=ddate, acute=T)     - Count the number of unique hospital episodes (epi variable in DAD)  1. Sedative-hypnotic harmful use or dependence n (%), see supplement 1 in the 730 days prior (excluding index date) to cohort entry 2. Stimulant harmful use or dependence n(%),see supplement1, in the 730 days prior (excluding index date) to cohort entry 3. ACGs (co-morbidity), see supplement 1, in the 730 days prior (excluding index date) to cohort entry |  |
| **Other Variables** |  |  |

| Analysis Plan and Dummy Tables (expand/modify as needed) *(please ensure the analysis plan is outlined with dummy tables (can be a separate document)*  *and clear specification of exposures / outcomes / covariates for each model)* | |
| --- | --- |
| **Descriptive Tables (insert or append dummy tables), e.g.:** | |
| **Table 1. Baseline characteristics stratified by sex and compared using standardized differences.**  **Descriptive information**  **-provide range for duration of prescription at index and number of benzodiazepines prescribed at index**  **-provide a histogram of individuals initiating benzodiazepines at baseline age**  **-provide distribution of drug names at index overall and by sex. Please order table highest % to lowest overall.** | |
| **Table 2. Survival estimates from Kaplan-Meier curves overall, and stratified by sex.** Calculate median time to discontinuation, overall and stratified by sex. (Provide based on KM curve) Present log-rank test for curves by sex. Construct KM in excel or provide data for same. | |
| **Statistical Model(s)** | |
| **Type of model** | Cox proportional hazards model |
| **Primary independent variable** | Objective 1: Sex  Objective 2: Days supplied |
| **Dependent variable** | discontinuation |
| **Covariates** | Age  Rurality  Income quintile  Sedative-hypnotic harmful use or dependence  Alcohol use disorder  OAT in last 6 months (180 days)  ACG  Discipline of prescriber  Anxiety disorders and Mood disorders (outpatient codes)  Psychotic disorders (outpatient codes)  Insomnia  Test PH assumptions using LLS curves |
|  | Provide the following 3 models:   1. Please check for collinearity among the secondary exposures of interest (prescription-related variables: number of prescriptions, duration of initial prescription, short vs. long. Vs. both, mean daily dose). If they are not collinear, please provide cox proportional hazards model with primary independent variale (sex), prescription-related variables, and dependent variable (discontinuation) and above covariates. 2. Assuming they are not co-linear, please also construct a second cox model with primary (days supplied) and secondary exposures of interest (prescription-related variables: number of prescriptions, type of prescription (short vs. long. Vs. both), mean daily dose)and the outcome discontinuation, controlling for above covariates. Please add sex as an interaction term in that model. 3. If sex as an interaction term is significant, please provide a third cox model with primary (days supplied) and secondary exposures of interest (prescription-related variables: number of prescriptions, type of prescription (short vs. long. Vs. both), mean daily dose)and outcome discontinuation, controlling for above covariates, stratified by sex. |
| **Sensitivity analysis** | Conduct same analyses using a 2.0x days supply and mingrace of 60 days in the NMScohort macro.  Conduct sensitivity analysis with primary cohort using dosing DME conversion (supplement 1) |

| **Post hoc sensitivity analyses:** Provide Objective 2 model stratified by time period early vs. late (2013-2016 vs. 2017-2020) |
| --- |
| Provide Objective 2 model using first episode in the accrual period. |
| Include all first and recurrent episodes as of July 1, 2014 allowing a 2 year look back. Run a PWP-TT model overall and stratified by episode number |
