# Supplementary material for: Association between initial benzodiazepine prescribing patterns and time to benzodiazepine discontinuation: A population-based retrospective cohort study
Source: PLoS Med. 2026 Jun 18;23(6):e1005126. doi: 10.1371/journal.pmed.1005126 (PMC13278425; doi:10.1371/journal.pmed.1005126)
Supplement: S1 STROBE Checklist — STrengthening the Reporting of OBservational studies in Epidemiology (STROBE) Statement – checklist of items that should be included in reports of observational studies, licensed under CC BY 4.0. von Elm E, Altman DG, Egger M, Pocock SJ, Gøtzsche PC, Vandenbroucke JP; STROBE Initiative. The Strengthening the Reporting of Observational Studies in Epidemiology (STROBE)statement: guidelines for reporting observational studies. PLoS Med. 2007 Oct 16;4 (10):e296. PMID: 17941714. (DOCX) [file pmed.1005126.s003.docx]

S1 STROBE Statement

|  | Item No. | Recommendation | Page  No. | Relevant text from manuscript |
| --- | --- | --- | --- | --- |
| **Title and abstract** | 1 | (*a*) Indicate the study’s design with a commonly used term in the title or the abstract | Title | Title: “Association between initial benzodiazepine prescribing patterns and time to benzodiazepine discontinuation: A population-based retrospective cohort study” |
|  |  | (*b*) Provide in the abstract an informative and balanced summary of what was done and what was found | Abstract section |  |
| Introduction | | | |  |
| Background/rationale | 2 | Explain the scientific background and rationale for the investigation being reported | Introduction |  |
| Objectives | 3 | State specific objectives, including any prespecified hypotheses | Final paragraph of the Introduction | “We therefore set out to understand the association of initial prescription characteristics with time to discontinuation of benzodiazepines in adults 18 years or older in Ontario, Canada, and given the disproportionate impact of benzodiazepine prescribing on women, whether this relationship differed by sex.” |
| Methods | | | |  |
| Study design | 4 | Present key elements of study design early in the paper | Methods section “Study Design and Setting” | “We conducted a retrospective population-based cohort study using linked administrative health data from the province of Ontario, Canada.” |
| Setting | 5 | Describe the setting, locations, and relevant dates, including periods of recruitment, exposure, follow-up, and data collection | Methods section “Cohort Creation” | “We included adults aged 18 or older with a new prescription for oral benzodiazepines (excluding z-drugs which are not uniformly available in the NMS database) between January 1, 2013 and December 31, 2020, defined as no benzodiazepine prescription in the 182 days prior to cohort entry.” |
| Participants | 6 | (*a*) *Cohort study*—Give the eligibility criteria, and the sources and methods of selection of participants. Describe methods of follow-up  *Case-control study*—Give the eligibility criteria, and the sources and methods of case ascertainment and control selection. Give the rationale for the choice of cases and controls  *Cross-sectional study*—Give the eligibility criteria, and the sources and methods of selection of participants | Methods section “Cohort Creation” |  |
|  |  | (*b*) *Cohort study*—For matched studies, give matching criteria and number of exposed and unexposed  *Case-control study*—For matched studies, give matching criteria and the number of controls per case | n/a |  |
| Variables | 7 | Clearly define all outcomes, exposures, predictors, potential confounders, and effect modifiers. Give diagnostic criteria, if applicable | Methods section “Exposures”, “Outcomes”, “Covariates”, S1 Table 4,5 |  |
| Data sources/ measurement | 8* | For each variable of interest, give sources of data and details of methods of assessment (measurement). Describe comparability of assessment methods if there is more than one group | Methods section “Exposures”, “Outcomes”, “Covariates”, S1 Table 4,5 |  |
| Bias | 9 | Describe any efforts to address potential sources of bias | Methods section, “Sensitivity Analyses” Limitations Section |  |
| Study size | 10 | Explain how the study size was arrived at | Figure 1 |  |

Continued on next page

| Quantitative variables | 11 | Explain how quantitative variables were handled in the analyses. If applicable, describe which groupings were chosen and why | Methods section ‘Exposures’ | “In all cases, categories were determined to align with clinical practice and to support clinical interpretation of results.” |
| --- | --- | --- | --- | --- |
| Statistical methods | 12 | (*a*) Describe all statistical methods, including those used to control for confounding | Methods section “Analysis” |  |
|  |  | (*b*) Describe any methods used to examine subgroups and interactions | Methods section “Analysis” |  |
|  |  | (*c*) Explain how missing data were addressed | Methods section, final paragraph | “There was missingness in only one variables (specialty of physician prescriber) and this was grouped separately.” |
|  |  | (*d*) *Cohort study*—If applicable, explain how loss to follow-up was addressed  *Case-control study*—If applicable, explain how matching of cases and controls was addressed  *Cross-sectional study*—If applicable, describe analytical methods taking account of sampling strategy | n/a |  |
|  |  | (*e*) Describe any sensitivity analyses | Methods section “Analysis” | “Multiple sensitivity analyses were conducted to assess the robustness of our findings…” |
| Results | | | | |
| Participants | 13* | (a) Report numbers of individuals at each stage of study—eg numbers potentially eligible, examined for eligibility, confirmed eligible, included in the study, completing follow-up, and analysed | Figure 1 |  |
|  |  | (b) Give reasons for non-participation at each stage | Figure 1 |  |
|  |  | (c) Consider use of a flow diagram | Figure 1 |  |
| Descriptive data | 14* | (a) Give characteristics of study participants (eg demographic, clinical, social) and information on exposures and potential confounders | Results section, 2^nd^ paragraph, Table 1 | “Baseline characteristics overall and stratified by sex are presented in Table 1. Overall, the median age at index was 53 (IQR 38-67), and 62.6% of included episodes occurred in females. Males were more likely to have a diagnosis of alcohol use disorder and any substance use disorder while females were more likely to have a mood or anxiety disorder diagnosis. Lorazepam was the most commonly prescribed benzodiazepine on index overall (63.8%), followed by clonazepam (17.2%) and diazepam (5.75%) (Table 1). Males were more likely to be prescribed diazepam (9.2%) while females were more likely to be prescribed lorazepam (66.9%).” |
|  |  | (b) Indicate number of participants with missing data for each variable of interest | Table 1 |  |
|  |  | (c) *Cohort study*—Summarise follow-up time (eg, average and total amount) | Results section, second paragraph |  |
| Outcome data | 15* | *Cohort study*—Report numbers of outcome events or summary measures over time | *Figure 1, Tables 2-4, Results section* |  |
|  |  | *Case-control study—*Report numbers in each exposure category, or summary measures of exposure |  |  |
|  |  | *Cross-sectional study—*Report numbers of outcome events or summary measures |  |  |
| Main results | 16 | (*a*) Give unadjusted estimates and, if applicable, confounder-adjusted estimates and their precision (eg, 95% confidence interval). Make clear which confounders were adjusted for and why they were included | Results section |  |
|  |  | (*b*) Report category boundaries when continuous variables were categorized | n/a |  |
|  |  | (*c*) If relevant, consider translating estimates of relative risk into absolute risk for a meaningful time period | n/a |  |

Continued on next page

| Other analyses | 17 | Report other analyses done—eg analyses of subgroups and interactions, and sensitivity analyses | Results section |  |
| --- | --- | --- | --- | --- |
| Discussion | | | | |
| Key results | 18 | Summarise key results with reference to study objectives | Discussion section, 1^st^ paragraph | “In this analysis of nearly 2 million benzodiazepine treatment episodes, after adjusting for potential confounding variables, several initial benzodiazepine prescribing patterns were associated with time to benzodiazepine discontinuation. Specifically, longer initial index prescriptions were strongly associated with a reduced likelihood of benzodiazepine discontinuation, a finding that was replicated in sex stratified analyses, and in all sensitivity analyses. Furthermore, receipt of long-acting benzodiazepines at initiation, multiple benzodiazepine types initiated, two or more benzodiazepines initiated, were consistently associated with prolonged benzodiazepine use across the analyses. Across the various models, the aHR of mean daily dose was close to 1, suggesting mean daily dose is a less important initial prescribing factor. Effect sizes were largest for longer initial prescriptions, and 2 or more benzodiazepines (compared with 1) initiated suggesting these may be clinically more important factors associated with long-term use.” |
| Limitations | 19 | Discuss limitations of the study, taking into account sources of potential bias or imprecision. Discuss both direction and magnitude of any potential bias | Discussion paragraph 8 |  |
| Interpretation | 20 | Give a cautious overall interpretation of results considering objectives, limitations, multiplicity of analyses, results from similar studies, and other relevant evidence | Discussion, last paragraph |  |
| Generalisability | 21 | Discuss the generalisability (external validity) of the study results | Discussion paragraph 8 | “This was a large population-based study in Canada’s most populous province, capturing all outpatient prescriptions for benzodiazepines across the province, regardless of payer, and linked administrative data reflecting all health service utilization in a publicly-funded system which supports the generalizability of our findings.” |
| Other information | |  | | |
| Funding | 22 | Give the source of funding and the role of the funders for the present study and, if applicable, for the original study on which the present article is based |  | See funding statement, not included in manuscript per submission instructions. |

*Give information separately for cases and controls in case-control studies and, if applicable, for exposed and unexposed groups in cohort and cross-sectional studies.

**Note:** An Explanation and Elaboration article discusses each checklist item and gives methodological background and published examples of transparent reporting. The STROBE checklist is best used in conjunction with this article (freely available on the Web sites of PLoS Medicine at http://www.plosmedicine.org/, Annals of Internal Medicine at http://www.annals.org/, and Epidemiology at http://www.epidem.com/). Information on the STROBE Initiative is available at www.strobe-statement.org.
